# Store-Operated Calcium Entry in Skeletal Muscle: What Makes It Different?

**DOI:** 10.3390/cells10092356

**Published:** 2021-09-08

**Authors:** Elena Lilliu, Stéphane Koenig, Xaver Koenig, Maud Frieden

**Affiliations:** 1 Center for Physiology and Pharmacology, Department of Neurophysiology and Pharmacology, Medical University of Vienna, 1090 Vienna, Austria; elena.lilliu@meduniwien.ac.at; 2 Department of Cell Physiology and Metabolism, University of Geneva, 1201 Geneva, Switzerland; stephane.konig@unige.ch

**Keywords:** skeletal muscle, store-operated Ca^2+^ entry, STIM, Orai, phasic SOCE, SOCE pharmacology, Ca^2+^ entry sites

## Abstract

Current knowledge on store-operated Ca^2+^ entry (SOCE) regarding its localization, kinetics, and regulation is mostly derived from studies performed in non-excitable cells. After a long time of relative disinterest in skeletal muscle SOCE, this mechanism is now recognized as an essential contributor to muscle physiology, as highlighted by the muscle pathologies that are associated with mutations in the SOCE molecules STIM1 and Orai1. This review mainly focuses on the peculiar aspects of skeletal muscle SOCE that differentiate it from its counterpart found in non-excitable cells. This includes questions about SOCE localization and the movement of respective proteins in the highly organized skeletal muscle fibers, as well as the diversity of expressed STIM isoforms and their differential expression between muscle fiber types. The emerging evidence of a phasic SOCE, which is activated during EC coupling, and its physiological implication is described as well. The specific issues related to the use of SOCE modulators in skeletal muscles are discussed. This review highlights the complexity of SOCE activation and its regulation in skeletal muscle, with an emphasis on the most recent findings and the aim to reach a current picture of this mesmerizing phenomenon.

## 1. Overview of the Muscle Structure and Ca^2+^ Handling

**Figure 1:**
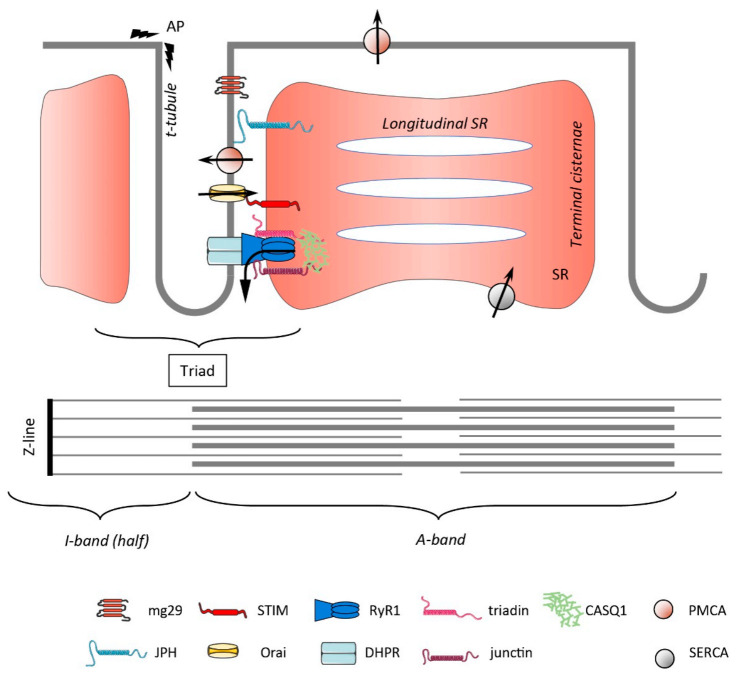
Schematic representation of the main components of the triad and the contractile apparatus. The thick and thin lines represent the myosin and actin filaments, respectively. *AP*—action potential, *mg29*—mitsugumin 29, *JPH*—junctophilin, *RyR1*—ryanodine receptor 1, *DHPR*—dihydropyridine receptor, *CASQ1*—calsequestrin 1, *PMCA*—plasma membrane Ca^2+^ ATPase, *SERCA*—sarco-endoplasmic reticulum Ca^2+^ ATPase.

Skeletal muscle fibers are very large multinucleated cells that are formed by the fusion of precursor cells, namely, myoblasts. They have a highly organized internal architecture, with most of the cell volume being occupied by the contractile elements, mainly actin and myosin, grouped as myofibrils. Sarcomeres are the functional units of the contractile apparatus, delineated by the Z-lines, which are oriented perpendicular to the long axis of the fibers. Within the sarcomeres, the strict arrangement of actin and myosin gives rise to the typical striated pattern of skeletal muscles, with alternating A and I bands, where the Z-line is found in the middle of the I-band (Figure 1). Each myofibril is surrounded by the sarcoplasmic reticulum (SR), which is a specialized region of the endoplasmic reticulum (ER) forming an interconnected network with a high Ca^2+^ buffering capacity (rev in [1]). Twice per sarcomere, the plasma membrane has deep invaginations called the t-tubules. Remarkably, it is estimated that the t-tubules encompass around 80% of the plasma membrane [2]. Facing each side of the t-tubules are enlargements of the SR forming the junctional SR (jSR; also called the terminal cisternae, TC) that, together with the t-tubules, compose the triad. The jSR is enriched in the acidic Ca^2+^ buffer calsequestrin (CASQ) and is in continuity with the longitudinal SR (lSR), which harbors a high density of sarco-endoplasmic reticulum Ca^2+^ ATPase (SERCA) pumps (rev in [3]). Fast and slow muscle fibers, so named according to their kinetics of contraction, compose a muscle. These fiber types differ in many aspects, among them, the size and buffering capacity of the SR, which are larger in fast fibers, together with a greater SERCA activity (rev in [4]). 

Excitation–contraction (EC) coupling depicts the succession of events leading to cytosolic Ca^2+^ elevation and, eventually, skeletal muscle contraction. It is initiated by the release of acetylcholine from the motor neuron nerve, which binds to its cognate ionotropic receptors at the neuromuscular junction (NMJ). The resulting cation influx depolarizes the plasma membrane (PM), activates Na_v_1.4 voltage-gated sodium channels, and triggers action potentials (APs) that propagate along the PM and into the t-tubules of skeletal muscle fibers. Within the t-tubules, the AP is sensed by L-type Ca_v_1.1 channels, which are also called dihydropyridine receptors (DHPRs). Through physical interaction, the change in DHPR conformation is transmitted to the RyR1 Ca^2+^ channel, which is located in the membrane of the jSR. The opening of RyR1 leads to an explosive and simultaneous release of Ca^2+^ from the SR into the cytoplasm across sarcomeres, initiating muscle contraction. After stimulation, Ca^2+^ clearance from the cytosol is accomplished by the SERCA pumps, which mediate the re-uptake of Ca^2+^ into the SR store. Due to the very brief muscle AP and the intrinsic biophysical properties of Ca_v_1.1, there is virtually no Ca^2+^ flux through these channels during membrane depolarization (rev in [5]). Hence, EC coupling *per se* is a process that does not require extracellular Ca^2+^ entry, in contrast to cardiac muscle, where Ca^2+^ influx is mandatory for the RyR (RyR2 in that case) to open and permit muscle contraction. The non-requirement of Ca^2+^ entry for EC coupling in part explains the delayed interest for SOCE in skeletal muscle.

SOCE is a ubiquitous mechanism that allows Ca^2+^ to enter the cells in response to a decrease in the ER/SR Ca^2+^ content. This peculiar mechanism was originally described in 1986 in the pioneering work of J. Putney on salivary glands, where this mechanism was called capacitive Ca^2+^ entry at that time [6]. After 20 years of intense research to elucidate the molecular components of this Ca^2+^ entry, a handful of studies uncovered the proteins supporting SOCE, namely, the stromal interaction molecule 1 (STIM1; [7,8,9]) and Orai1 [10,11,12]. STIM1 is a single-pass transmembrane protein that is localized on the ER that binds Ca^2+^ via its luminal EF-hand motifs. Upon store depletion, the unbinding of Ca^2+^ leads to STIM1 oligomerization and translocation toward the plasma membrane (PM). The unfolding of the protein exposes a polybasic region at the C-terminal end of the molecule, which promotes its recruitment/stabilization at the PM via the binding of phosphoinositides [13,14,15]. Other exposed key residues of STIM1 within the CAD (CRAC activation domain), allow the gating of Orai1 and eventually Ca^2+^ entry. The whole process from ER Ca^2+^ depletion to the activation of Ca^2+^ entry takes tens of seconds, mainly as a result of STIM1 translocation to the PM (rev in [16]). The translocation toward the PM is associated with a profound remodeling of the ER, forming thin and elongated ER cisternae (cortical ER (cER)) visible in electron micrographs [17,18]. STIM2, the other member of the STIM family, has a higher affinity for phosphatidylinositol 4,5-bisphosphate (PiP_2_) due to a modified polybasic domain [19,20]. In addition, the Ca^2+^ affinity of STIM2 (K_d_ for Ca^2+^ binding around 400 µM) is lower than that of STIM1 (K_d_: 200 µM), making STIM2 a regulator of the basal cytosolic Ca^2+^ concentration and the ER Ca^2+^ level (rev in [21]). The PM Ca^2+^-selective Orai channel comprises three members, namely, Orai1–3. The current flowing through Orai, called Ca^2+^ release activated Ca^2+^ current (I_CRAC_), has been known since the nineties (well before the molecular identification of Orai channels), and is nowadays very well characterized. Its peculiar electrophysiological signature comprises a tiny unitary conductance in the fS range, which precludes single-channel recording, a strong inward rectification with a very positive reversal potential highlighting its Ca^2+^ selectivity, and a complex regulation both by intra- and extracellular Ca^2+^ concentration [22]. In particular, I_CRAC_ undergoes fast and slow Ca^2+^-dependent inactivation (CDI) that are proposed to prevent excessive Ca^2+^ entry and, thus, a potential overload that would be detrimental for cell function [23,24].

In contrast to the great interest in SOCE in non-excitable cells, studies on skeletal muscle SOCE failed to appear for several years following the initial description by J. Putney. Several reasons could account for this relative disinterest: as mentioned, EC coupling *per se* does not require an external Ca^2+^ influx and skeletal muscle contraction can occur, at least for a while, in a medium devoid of Ca^2+^ [25]. Furthermore, once Ca^2+^ is released from the SR into the cytosol, at least during moderate muscle activity, almost all of it is pumped back into the SR with basically no loss across the PM (Ca^2+^ flux across the PM is orders of magnitude smaller than Ca^2+^ flux across the SR membrane; [26]). Hence, it was assumed that there was no need for external Ca^2+^ entry to replenish the Ca^2+^ stores in skeletal muscle. More technically and linked to muscle size, the classical protocol that is used to activate SOCE, i.e., blocking the SERCA pumps in the absence of external Ca^2+^ to passively deplete the stores, does not result in a massive store depletion in skeletal muscle. Indeed, the SR Ca^2+^ store is a large compartment with a very high Ca^2+^-buffering capacity. In addition, the SR membrane in skeletal muscle is less leaky compared to non-excitable cells, impeding SERCA blockage-induced store depletion [27,28], and thus SOCE. Lastly, electrophysiological recordings of I_CRAC_ in skeletal muscle are scarce, with only two papers reporting such a current in myotubes [29,30]. Actually, the group of B. Allard claimed that in skeletal muscle, I_CRAC_ is below the limit of detection, even using the silicon clamp approach, which allows for accurate voltage clamping of the membrane [31,32]. Hence, this strongly limits the knowledge we have on the biophysical properties of the skeletal muscle SOCE current. For all these reasons, the interest in SOCE in the muscle system really emerged only after the first work clearly revealed this pathway in 2001 [33]. In this study performed on isolated fibers from *extensor digitorum longus* (EDL), the SR was depleted by successive applications of high K^+^ solution in a Ca^2+^-free medium, followed by a treatment to block the SERCA pumps. The subsequent Ca^2+^ re-addition replenished the stores, which was indicative of SOCE having taken place. Mn^2+^ quenching experiments further confirmed the activation of SOCE. With the identification a few years later of the proteins supporting SOCE and the finding that mutations in STIM1 or Orai1 are associated with muscle pathologies, the field gained much attention. Indeed, loss-of-function mutations of STIM1 and Orai1 lead, besides a severe immunodeficiency, to congenital myopathy, which is characterized by hypotonia and reduced muscle endurance. Gain-of-function mutations in SOCE molecules also result in progressive muscle weakness, known as tubular aggregate myopathy (TAM) syndrome (rev in [34]).

## 2. Localization and Role of SOCE Molecules in Cells with “Constrained” Architecture

### 2.1. Localization and Movements of STIM and Orai 

Under resting conditions, STIM1 is diffusely distributed in the ER membrane in a folded conformation and undergoes comet-like movement. This movement, which is observed with fluorescently tagged STIM1, results from its interaction with the microtubule plus end-binding protein EB1 [35,36] and follows the elongation of microtubules. Upon store depletion, STIM1 detaches from EB1 [35], at least partially [36], and translocates toward the PM. The movement is accompanied by STIM1 oligomerization, forming characteristic punctae structures at the PM. Thanks to the polybasic domain of the protein, STIM1 binds to PiP_2_ phospholipids of the PM, and eventually traps and gates Orai channels, allowing for Ca^2+^ entry. STIM1 binding to EB1 is not required for SOCE to take place; on the contrary, it was proposed that it slows down the localization of STIM1 at the ER-PM and thus prevents excessive Ca^2+^ entry, potentially leading to Ca^2+^ overload [36]. The translocation of STIM1 also implies a reorganization of the ER with the formation of cortical ER sheets found at a distance of around 11–12 nm from the PM [17,18]. In skeletal muscle, the distance between the jSR and the t-tubules, measured as being 12–15 nm [37], is compatible with a proper interaction between STIM1 and Orai1. However, what do we know about STIM1 localization in muscle cells? Does the highly ordered internal architecture of skeletal muscle allow for STIM1 movement and its PM translocation?

**Figure 2:**
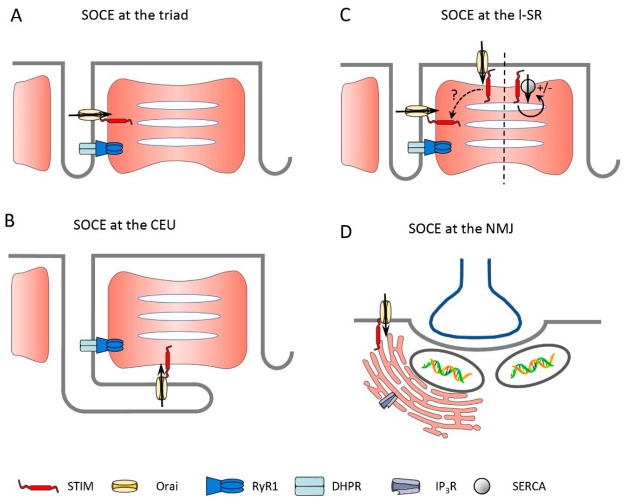
Schematic representation of possible sites of SOCE in skeletal muscle. (**A**) SOCE at the triad is well supported and the most described in the literature. STIM and Orai are expressed in the triad membranes and respective SOCE was confirmed. (**B**) SOCE at the CEU is well described as a newly formed structure that is induced by strenuous exercise. STIM and Orai are expressed at the CEUs but the respective SOCE Ca^2+^-flux has not yet been demonstrated to date. (**C**) STIM at the lSR moves to the triad upon activation or activates Orai at the PM. It was also reported to modulate the activity of the SERCA pump. (**D**) Localization at the NMJ remains speculative for the time being.

Immunostaining of STIM1 in mice hindlimb muscle showed a clear striated pattern, colocalizing with RyR1, which is indicative of STIM1 being at the triad. Biochemical analysis after membrane fractionation also revealed STIM1 in the lSR [29]. The triad localization of STIM1 was confirmed on *flexor digitorum brevis* (FDB) fibers, together with its colocalization with Orai1. Wei-LaPierre et al. claimed that these colocalized proteins did not form a SOCE complex until cells were treated with thapsigargin (Tg, a SERCA blocker), based on a bimolecular fluorescence complementation assay [38]. However, their conclusion was based on an uncalibrated assay that potentially fails to detect an assembly if the fraction of STIM1–Orai1 coupling is very low at rest (discussed in [39]). STIM1 and Orai1 localization at the triad is not surprising considering that the t-tubules comprise about 80% of the plasma membrane in skeletal muscle and thus provide the largest access to extracellular space. The physiological function of the smaller proportion of STIM1 found at the lSR is not known, but it might represent a “reserve” pool of the protein that can be mobilized and move to the jSR to further increase SOCE whenever required. Moreover, this “longitudinal STIM1” pool could gate Orai1 at the PM for the lSR around the more peripheral myofibrils (Figure 2C) or could serve other functions unrelated to Ca^2+^ entry, such as enhancing SERCA1 activity [40]. Strong staining of STIM1 at the lSR (the A–I band junction) was recently reported [41], the functional consequence of which will be discussed in Section 2.2. Orai1 was also recently proposed to be present in two different pools within the muscle fiber. One pool of Orai1 is permanently associated with STIM1 and is responsible for fast Ca^2+^ entry being activated during EC coupling. Another pool is not in close proximity to STIM1 and would be recruited more slowly in case of substantial Ca^2+^ store depletion, which is typically induced by SERCA inhibition and would be linked to a slower process of Ca^2+^ refilling [42]. The two pools of Orai1 are defined functionally but are not distinguished by a separated localization within the cells, with all Orai1 being observed at the triad. Further studies would be needed to confirm this finding and better define its physiological implications.

What about the mobility of STIM1 in skeletal muscle? It is known that proteins located at the jSR are much less mobile than those found at the lSR. This was convincingly shown using FRAP (fluorescence recovery after photobleaching) experiments on differentiated myotubes, with triadin being the least mobile protein, followed by RyR1 and junctin [43]. Before triad formation occurs, however, those proteins are mobile, as both the diffusion rate and the mobile fraction of the proteins are elevated. By comparison, proteins of the lSR, such as SERCA2, were shown to retain high mobility in differentiated myotubes [43]. Hence, during myotube formation, the mobility of proteins that are eventually localized at the triad decreases; the mechanism ensuring this localization remains uncertain (rev in [37]). In a recent elegant paper, Sébastien et al. [44] revisited triad protein mobility and showed, using a photoactivatable form of the protein, that a small fraction of triadin (also called Trisk 95), which is a single-pass transmembrane protein of the triad, is mobile and can move in and out of the triad. Once at the triad, and in line with the report of Cusimano [43], the mobility of triadin is greatly reduced. The authors also identified the transmembrane (TM) domain of Trisk 95 as an essential motif that retains the protein at the jSR [44]. It would be very informative to know whether STIM1 diffuses in the SR membrane just as it does in non-muscle cells, whether its diffusion is limited like for Trisk 95, or whether it is permanently retained at the triad. Two main arguments are in favor of STIM1 being retained at the triad (at least for a large part of the pool): First, the very fast kinetics of SOCE activation in muscle cells is incompatible with the translocation of STIM1 upon activation (see Section 3 about phasic SOCE). The second aspect is related to the site of store depletion during EC coupling. Indeed, SR Ca^2+^ depletion occurs mainly at the jSR [45,46] and thus for STIM1 to sense this local Ca^2+^ decrease, the protein must be located there as well. One should also consider that STIM1 can be activated following other types of stimulation, for instance, after IP_3_ receptor (IP_3_R)-induced Ca^2+^ release. However, while this pathway seems to be relevant in developing muscle, there are controversies about its existence in adult muscle. Blaauw et al. [47] claimed that no IP_3_R-induced Ca^2+^ release could be recorded in adult skeletal muscle after using different approaches, such as direct IP_3_ injection, IP_3_ uncaging, or stimulation by agonist-induced IP_3_ formation. In contrast, IP_3_R1 was reported to be localized at the lSR, colocalizing with the Z-line in differentiating myotubes [43], and the group of Jaimovich described a small amount of IP_3_R-induced Ca^2+^ release that takes place preferentially around the nuclei and induces transcriptional activity [48,49]. More recently the same group showed that mitochondria Ca^2+^ uptake, resulting from Ca^2+^ release, is partially sensitive to IP_3_R blockers [50]. Hence, it remains to be defined whether STIM1 could potentially sense SR Ca^2+^ decrease after IP_3_R-induced Ca^2+^ release and thus induce SOCE in muscle fibers. Related to the IP_3_R, the ER compartment found around the nuclei and in continuity with the SR localized around the myofibrils and implicated in EC coupling [1] should be considered. Indeed, a high density of IP_3_R was found around nuclei, in particular, those at the NMJ [51,52], and one can speculate that a subset of STIM molecules is also present under the NMJ (Figure 2D) and could be implicated in the specific Ca^2+^ signals that are required for the proper formation of the NMJ [52]. Thus, several open questions related to the localization of STIM1 molecules at different places in adult muscle fibers and the putative mobility of STIM1 remain to be addressed.

### 2.2. Regulation of SOCE in Skeletal Muscle

The triad was the first ER/SR-PM contact site described [53]. Nowadays, many membrane contact sites (MCS), which are defined as a tight apposition between the ER membranes and other organelle’s membrane or PM, are identified within cells, with the ER-PM region being the best characterized [54]. The skeletal muscle triad is intensively studied in terms of the formation, molecular composition, and regulation of Ca^2+^ release. The DHPR and the RyR1, which are abundantly expressed at the triad, are the two key Ca^2+^ channels supporting EC coupling, but several other proteins are located there, providing a structural function or acting as regulators of Ca^2+^ handling. We will here only briefly mention the most relevant of these and highlight their putative function as direct or indirect SOCE modulators (Figure 1). 

Junctophilin 1 and 2 (JPH1 and JPH2) are anchored in the SR membrane and possess a large N-terminal hydrophobic region that binds phospholipids in the PM, making JPHs key proteins to couple the SR to the t-tubule membrane. Indeed, knockdown of both JPHs leads to a malformation of the triad that is associated, among other defects, with reduced SOCE [55,56]. Interestingly, neither RyR1 nor DHPR fulfills a structural function, as shown by animal models lacking one or the other protein, that maintain a proper triad formation [57,58]. With RyR1 being the most important Ca^2+^ release channel of skeletal muscle, it is indirectly coupled to SOCE activation and, in addition, SOCE (induced by SERCA inhibition) is reduced in myotubes from mice lacking RyR1 [59]. Triadin is a family of SR-associated proteins that originate from the alternative splicing of the TRDN gene [60]. In skeletal muscle, three members of the family are expressed, namely, Trisk 95, Trisk 51, and Trisk 32, with the first two being expressed the highest. Trisk 95 is associated with RyR1 and CASQ1, and by promoting membrane deformation, it favors the interaction between RyR1 and DHPR and thus EC coupling (rev in [61]). Junctin and junctate are two ER/SR proteins that are generated by a complex splicing of the same gene, which also encodes humbug and aspartyl β-hydroxylase (rev in [62]). Junctin expression is restricted to cardiac and skeletal muscle, where it associates with RyR1, triadin, and CASQ1. It was shown to modulate the open probability of RyR1 but also to play a role in the SR Ca^2+^ storage size [63]. In contrast, junctate is more broadly expressed and is a high-capacity Ca^2+^ binding protein [64]. Overexpressing junctate specifically in skeletal muscle resulted in an increased SR Ca^2+^ content and an enhanced caffeine-induced Ca^2+^ release, but also an increased Ca^2+^ entry after store depletion [65]. A few years later, it was reported that junctate indeed facilitates the recruitment of STIM1 to the ER-PM junction. This mechanism was thus postulated as the third one favoring the localization of STIM1 at the PM upon store depletion, in addition to phospholipids and Orai binding [66]. These experiments were conducted in T cells, but one can reasonably hypothesize that such an association between STIM1 and junctate also takes place in skeletal muscle to promote SOCE. CASQ1 is highly expressed at the jSR of skeletal muscle and was reported to have an inhibitory effect on SOCE [67], which was confirmed in CASQ1 knockdown experiments [68]. Later, CASQ1 was indeed shown to interact with STIM1, preventing its binding to Orai1 and thus limiting SOCE [69,70]. Mitsugumin 29 (mg29) is a synaptophysin-like protein that is localized at the t-tubules [71]. Mg29^−/−^ animals presented structural defects, such as swollen t-tubules, vacuolization of the SR, and a misalignment of the triad, even if the triad is present [59,72]. These structural alterations were associated with a reduced SR Ca^2+^ content, together with a reduced/slower SOCE [59,73]. Functionally, these animals were more prone to fatigue compared with wild-type controls [59,74]. Interestingly, the decreased expression of mg29 with age was postulated to account for the reduced SOCE observed in old animals, despite normal levels of STIM and Orai [73]. However, the role of mg29 as a potential regulator of muscle SOCE was not confirmed by another study that was performed on adult tissue [75], and thus the involvement of mg29 as a SOCE modulator remains an open question. Furthermore, the observed reduction of SOCE in aged mice was not confirmed by others [76].

By analogy with proteins at the triad, the MCS associated with STIM and Orai contains proteins that are involved in the establishment of the contact and several SOCE regulators. Extended synaptotagmin (E-syt1) was shown to be recruited by SOCE and then served to stabilize the MCS [77] (rev in [54]). In contrast to the triad, where RyR1 and DHPR do not have a structural function, the MCS containing SOCE are formed by the recruitment of STIM1 to the PM and its binding to Orai1. Several SOCE-interacting proteins were reported, such as SOCE-associated regulatory factor (SARAF), STIM-activating enhancer (STIMATE), partner of STIM (POST), or CRAC regulator 2A (CRACR2A), which serve as fine regulators of Ca^2+^ entry (rev in [78]). To the best of our knowledge, however, none of these regulatory proteins were reported to modulate SOCE in skeletal muscles. Nevertheless, the lipid composition of the PM was proposed to negatively regulate Orai1, with two residues in the N-terminus of the channel being implicated directly or indirectly in this regulation [79]. Interestingly, the t-tubule membrane is about four times more enriched in cholesterol than the sarcolemma [80,81], potentially influencing Orai1 function. Furthermore, PiP_2_ content modulates SOCE. Indeed, among the regulatory SOCE mechanisms, one is the well-known prevention of Ca^2+^ overload via the slow Ca^2+^-dependent inactivation (CDI) of SOCE/I_CRAC_, which is in part linked to SARAF binding. It was reported that the accessibility of SARAF to Orai1 is associated with the movement of the SOCE complex from PiP_2_-poor to PiP_2_-rich regions upon activation [82]. Whether such regulation of SOCE takes place in skeletal muscle is not known and will be difficult to address due to the quasi impossibility of I_CRAC_ recordings in these cells [83]. Hence, compared to what is known about SOCE/I_CRAC_ regulation in non-excitable cells, little is known about skeletal muscle SOCE regulation. In particular, how Ca^2+^ concentration within the narrow space between the t-tubule and the jSR influences both the RyR1 channels and the SOCE process (including CDI) is so far not addressed but would be fundamental to understand.

Some years ago, a different mechanism of muscle SOCE activation was proposed by the group of F. Protasi, arguing that the high density of RyR1 molecules at the triad would hinder the diffusion of STIM1 to the same place [84]. Along this line, they reported that STIM1 is localized at the lSR, more specifically at the I-band [41]. Orai1, on the other hand, was found at the triad, as previously reported [38]. Unexpectedly, electron microscopy revealed that after high-intensity (HIT) exercise, the t-tubules were massively reorganized, and to a lesser extent, the SR too. Essentially, the t-tubules elongated and moved parallel to the lSR, resulting in the formation of numerous contacts between both membranes and the appearance of characteristic stacks. Hence, this finding would imply that in skeletal muscle, the plasma membrane (in that case, the t-tubule) moves toward STIM1 and not vice versa, as is observed in other cell types. These peculiar t-tubule structures, called Ca^2+^ entry units (CEUs), were also occasionally seen in muscles from non-exercised mice. Immunostaining confirmed that STIM1 and Orai1 co-localization was minimal at rest but significantly enhanced upon HIT exercise [41]. In a follow-up paper, the same group reported that these were reversible structures, even if it took several hours (>6 h) to revert [85]. Functionally the CEUs were associated with better resistance to fatigue. Indeed, when EDL muscles (ex vivo experiments) from animals that previously ran intensively for one hour (and thus had newly formed CEUs) were undergoing high-frequency stimulations, the force decline was less compared to muscles from animals that did not run beforehand. This “beneficial“ effect was gone in the presence of the SOCE blockers BTP2 or 2-APB [41], and also in muscles from Orai1^−/−^ animals, highlighting a role of Orai1-dependent SOCE in this effect [85]. In addition, the formation of the CEUs upon intense exercise was associated with an increased basal SOCE, an enhanced resting cytosolic Ca^2+^ concentration, and a decrease in total SR Ca^2+^ [85]. The authors hypothesized that the increased basal Ca^2+^ entry might be due to STIM2-induced SOCE rather than STIM1, but this remains to be determined. These observations raised an additional question about the beneficial effect of the formation of the CEUs, as increased basal SOCE is known to be detrimental for cells and, in particular, for skeletal muscle, as highlighted by the pathologies linked to gain-of-function mutations of STIM1 or Orai1 (rev in [86]). So far, nothing is known about the mechanism leading to such t-tubule rearrangement, nor whether this is a general way SOCE presents upon physiological muscle use. What was, however, recently shown was an enhanced CEUs in mice lacking CASQ1, which is the main SR Ca^2+^ buffer [87]. In these animals, the Ca^2+^ storage capacity was greatly reduced and correlated with higher SOCE capacity. In addition, the expression levels of STIM1, Orai1, and SERCA were elevated, further promoting an increased SOCE that would permit sustained Ca^2+^ release upon repetitive stimulations in a context of reduced SR Ca^2+^ stores [87]. Overall, the massive rearrangement of the t-tubules and the formation of CEUs is an interesting new way of considering SOCE in skeletal muscle; a potential regulatory mechanism that is entirely different from what takes place in non-excitable cells. It should, however, be stressed that there is so far no direct evidence that Ca^2+^ entry takes place at the CEUs. Importantly, the lSR has a greatly reduced RyR1 density [88] and the RyR1 density at the CEUs was not increased upon exercise [41,85]. Given the pivotal role of RyR1 in the activation of SOCE [30,39,59,89,90], this raises the question of how SOCE could be activated at these sites. While it is appealing to speculate that the observed increase in SOCE is causally linked to and not only correlated with the newly formed CEUs, it might also be that the observed increase in SOCE upon exercise [85] is caused by an altered SR Ca^2+^-buffering capacity and/or structural/functional changes within the triads. Thus, strikingly, knock out of key SR and triad proteins, namely, triadin/junctin [91], CASQ [87], and microtubule-associated protein 6 (MAP6 [92]), induce CEU structures that fully resemble those found after strenuous exercise [41,84]. Interestingly, alterations in t-tubular structure and SOCE were also reported in human muscle after heavy-load resistance exercise [93]. Thus, Cully et al. [93] described the formation of vacuoles within the longitudinal t-system upon high-force eccentric exercise. These vacuoles, which formed within several hours after exercise and seem to be reversible after a few days, could store large amounts of Ca^2+^ but were devoid of SOCE. The authors argued that the absence of SOCE in the longitudinal t-system compartment reflected the fact that the SR does not form junctions and that RyRs are absent in these regions. This would prevent the activation of SOCE, even if a respective SOCE protein machinery is present at these sites. It will require further studies to better define the physiological mechanisms that lead to the observed changes in the t-system architecture upon different forms of exercise, as well as the physiological consequences that are related to such alterations in muscle structure.

Based on the different studies on skeletal muscle SOCE, we can conclude that it is more than likely that SOCE is taking place at various locations within an adult muscle fiber. Obviously, it occurs at the triad (see also the chapter below) and potentially at the CEUs, possibly depending on the fiber type and the intensity of exercise. Other putative locations, which are, however, so far not supported by literature, are the PM in connection with the SR, but also with the ER around the NMJ, with the latter likely being involved in gene regulations (Figure 2).

## 3. Muscle-Specific Fast Activation Kinetics of SOCE

### 3.1. Using “Skinned” Fibers to Measure SOCE

Probably the largest fraction of SOCE is conducted across the transverse tubules of the tubular (t-) system membrane specifically and not the PM [32,83,94] nor the longitudinal tubules of the t-system [93]. The fine- and complex-branched structure of the t-system and its location entirely within the fiber’s body made it inaccessible for conventional electrophysiological approaches [83,95,96]. It needed the development of fluorescent measurements from within the t-system of skinned fibers to obtain most of our current knowledge on the Ca^2+^-handling properties of the t-system, including SOCE [97]. These fluorescent techniques proved superior to the classical approaches because they allowed for a better temporal and spatial resolution and greater sensitivity as, e.g., compared to SOCE measurements using Mn^2+^-quenching experiments. Importantly, they also allowed for studying SOCE simultaneously with SR Ca^2+^ release in physiological salt solutions, normal activation patterns, and functional SR proteins, which cannot be achieved in standard electrophysiological measurements [96]. Therefore, we briefly sketch the development here.

Mechanically skinned fibers were originally described by Natori in 1954 [98]. Using this technique, access to the intracellular space of the fiber is gained by physically rolling back the sarcolemma with fine forceps [99,100]. The procedure leaves the integrity of the SR and t-system intact. In addition, the t-system entry mouths, which are connected to the sarcolemma beforehand, seal off to form a closed compartment after skinning. Fluorescent dyes diffuse into the open t-system when applied in an extracellular buffer prior to skinning [101] and are trapped therein upon skinning [102] because of the induced sealing-off of the t-tubule mouths. The necessary protein machinery that is used to conduct EC coupling resides within the t-system and is not affected by the removal of the sarcolemma during the skinning procedure. Hence, EC coupling is preserved in such a preparation, with normal [Ca^2+^]_cyto_ transients [45,96,103,104] and force responses [103,105], as seen in intact fibers. Recently, low-affinity Ca^2+^-sensitive dyes (Rhod-5N, Fluo-5N, and Mag-indo-1) trapped in the sealed t-system were used to investigate t-system Ca^2+^ handling [94,95,102] and were used to measure SOCE in a quantitative manner [93,97].

Soon after the first description of SOCE in skeletal muscle by Kurebayashi and Ogawa [33], it became apparent that SOCE in muscle was markedly different from that observed in non-muscle cells, in particular regarding its fast activation kinetics. Using a fluorescent dye trapped in the t-system of a skinned fiber preparation allowed Launikonis et al. to monitor [Ca^2+^]_t-sys_ and derive a measure of SOCE during SR Ca^2+^ release [95]. SR Ca^2+^ release was induced by lowering the free cytosolic magnesium concentration ([Mg^2+^]_cyto_), which removed the Mg^2+^-dependent inhibition of the RyR1 and caused a cell-wide Ca^2+^-release [97,106,107,108]. Note that if not otherwise stated, [Ca^2+^] or [Mg^2+^] will always refer to the respective free ion concentrations. Under these conditions, SOCE was activated within one second upon exposure to low [Mg^2+^]_cyto_. This was the first demonstration of fundamental differences in skeletal muscle SOCE because it was at odds with the classical view of SOCE activation as observed in, e.g., immune cells: STIM oligomerization, recruitment of PM and ER contact sites, puncta formation, and activation of Orai channels. SOCE, as observed in these early experiments, activated way too fast to allow for such a complicated mechanism of activation. A refinement of the technique led to further insights. Thus, Edwards et al. demonstrated that SOCE in mouse EDL muscle even occurs on a millisecond timescale [109]. To isolate SOCE and avoid the activation of voltage-dependent currents, SR Ca^2+^ release was induced via the direct activation of the RyR1, again using conditions of low [Mg^2+^]_cyto_. Besides a cell-wide Ca^2+^ release, low [Mg^2+^]_cyto_ commonly induces the emergence of propagating Ca^2+^ waves across the preparation, a condition under which SR Ca^2+^ buffers are constantly depleted and refilled, and SOCE can be studied. A low-affinity Ca^2+^-sensitive dye loaded into the t-system of a skinned fiber allowed for the continuous monitoring of SOCE, which reported the respective activation of SOCE only 27 ms after SR Ca^2+^ release had occurred. This fast kinetics indicated a steep relationship between changes in [Ca^2+^]_SR_ and activation of SOCE and suggested a physical pre-coupling between STIM1 and Orai1 within the triad region to account for the observed fast kinetics [26,95]. This was in agreement with the localization of STIM1 and Orai1 within the triad regions [29,38] and that disruption of the triad structure by knockout of junctophilin [55,110] or exposure of the fiber to sustained high [Ca^2+^]_cyto_ [94,95,102] reduced/abolished SOCE. Moreover, these experiments showed that SOCE activated as the RyR1 began to release Ca^2+^ and that this occurred well before the Ca^2+^ inside the SR was significantly depleted. This was an important step forward compared to experiments that relied on completely depleted SR Ca^2+^ stores, which also showed a RyR dependence of SOCE (the SOCE amplitude was greatly reduced in the myotubes of RyR1/RyR3-deficient mice [59] and in dyspedic (lacking RyR1) myotubes [89]. Furthermore, the skeletal muscle I_CRAC_ current, as assessed using a whole-cell patch clamp, was reduced three-fold in RyR1-null myotubes and inhibited by 100 µM ryanodine [30]), but could not resolve the full dynamics linking the activation of SOCE to the opening of the RyR1. 

While these early experiments defined key hallmarks of SOCE in muscle, by tracking the t-system Ca^2+^ simultaneously with SR Ca^2+^ release triggered via direct stimulation of the RyR1 [95,109], it was of interest to determine whether SOCE could also be activated using voltage stimulation and thereby operate during single muscle twitches. A first hint that this was possible was given in 2009 when Launikonis et al. [96] reported on an AP-dependent Ca^2+^-influx that was independent of L-type Ca^2+^ channels and that they named AP-activated Ca^2+^ current (APACC). While the authors argued for an SOCE-independent mechanism at that time, in retrospect, their results surprisingly reflect several key features of phasic SOCE observed at low cytosolic buffering conditions (see below). Another finding was that the rate of fura-2 quenching by Mn^2+^ changed upon electrical burst stimulation in interosseous muscle fibers [32]. The observed increase in the Mn^2+^ quenching rate was attributed to an electrically silent pathway suggested to be again independent of L-type Ca^2+^ channel function [32].

### 3.2. How to Measure SOCE during EC Coupling?

**Figure 3:**
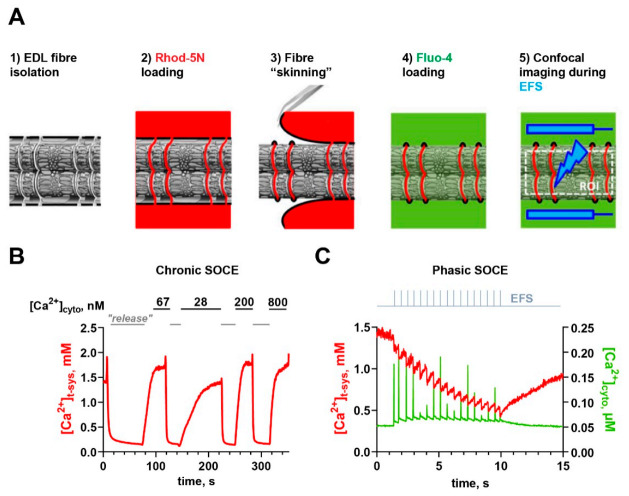
Two fluorescence-based techniques to measure SOCE in skinned skeletal muscle fibers. (**A**) Schematic representation of the different protocol steps to perform measurements of either chronic SOCE (steps 1–3) or phasic SOCE (steps 1–5). (1) Isolation of an EDL muscle and preparation of small muscle bundles under paraffin oil. (2) Incubation of muscle fibers with a low-affinity Ca^2+^-sensitive dye (e.g., Rhod-5N). (3) Skinning of individual fibers under paraffin oil traps Rhod-5N in the sealed t-system (to report [Ca^2+^]_tsys_) and opens access to the cytoplasm. (4) Transfer of a skinned muscle fiber with Rhod-5N trapped in the t-system to an experimental chamber filled with a physiological salt solution mimicking the muscle cytoplasm. Addition of a high-affinity Ca^2+^-indicator, e.g., Fluo-4, to enable the measurement of [Ca^2+^]_cyto_. (5) Mounting of the preparation on the stage of a confocal microscope that is able to perform fast image acquisition, e.g., being equipped with a resonant scanner, or a spinning disk system, and electrical field stimulation (EFS) via two platinum electrodes that are immersed in the bath solution positioned in parallel to the fibers’ long axis. (**B**) Measurement of *chronic* SOCE in a skinned fiber following steps 1–3 (e.g., [97]). Typical recording of [Ca^2+^]_t-sys_ derived from the calibrated fluorescence of Rhod-5N. The fiber is bathed in an internal salt solution containing 1 mM free Mg^2+^ and 67 nM free Ca^2+^; then, chronic SOCE is induced through direct activation of the RyR1 by exposing the fiber to a “release” solution containing 0 mM free Mg^2+^ and 30 mM added caffeine. Activation of chronic SOCE is seen as a steep depletion of [Ca^2+^]_t-sys_. The depletion is fully reversible as the t-system reloads with Ca^2+^ upon restoration of physiological [Ca^2+^]_cyto_ and [Mg^2+^]_cyto_. The sequence of depletion and re-uptake can be repeated at different [Ca^2+^]_cyto_ values. (**C**) Measurement of phasic SOCE in a skinned fiber following steps 1–5 in **A**. Typical phasic SOCE recording, consisting of the imaging of [Ca^2+^]_t-sys_ (left axis) and [Ca^2+^]_cyto_ (right axis) over time, as derived from a rat skinned EDL fiber. EFS triggers APs in the sealed t-system and concomitant SR Ca^2+^ release with respective [Ca^2+^]_cyto_ transients. Note that the high EGTA buffering leads to very sharp [Ca^2+^]_cyto_ transients, which are undersampled by the employed sampling rate, which results in a pseudo-modulation of [Ca^2+^]_cyto_ transient amplitudes. Phasic SOCE manifests as rapid depletion of [Ca^2+^]_t-sys_ upon each induced AP (with every EFS pulse). As for the measurements of chronic SOCE, [Ca^2+^]_t-sys_ recovers after the cessation of EFS due to the function of NCX and/or PMCA. Parts of the figure have been taken and modified from Koenig et al. [39]), published under CC BY 4.0 license.

A combination and advancement of different experimental approaches involving skinned muscle fibers [97,105], as described above, then led to the recent demonstration of SOCE during physiological activation patterns when SOCE was observed during single muscle twitches in skinned rat EDL fibers [39]. It was named *phasic* SOCE (pSOCE) to reflect the transient nature of the observed Ca^2+^ flux and to discriminate it from slower and longer-lasting forms of SOCE, referred to as *chronic* SOCE (cSOCE; [39]). The recording of pSOCE during AP-induced SR Ca^2+^ release was made possible by the simultaneous tracking of [Ca^2+^]_cyto_ and [Ca^2+^]_t-sys_ at high temporal resolution and fidelity using high-speed confocal microscopy and concomitant electric field stimulation in skinned muscle fibers. An overview of two important, fluorescence-based experimental paradigms for measuring SOCE using skinned skeletal muscle fibers is given in Figure 3.

One key point of the technique involves the use of high levels of cytosolic Ca^2+^ buffering with 10 mM EGTA [39,111]. This allowed us to isolate SOCE by reducing the [Ca^2+^]_cyto_ reached during an AP (see below). Moreover, it enabled calculating the amount of Ca^2+^ released during every muscle twitch and thereby allowed for determining the dependence of pSOCE on SR Ca^2+^ release [39]. Another key point was that the technique employed fluorescence averaging across the entire muscle fiber, which is possible because electrical field stimulation (EFS) triggers synchronous APs in every sarcomere. This significantly improved the signal-to-noise ratio, in particular regarding the weak Rhod-5N fluorescence emanating from within the t-system, which constitutes only a small fraction of the total fiber volume. The necessary fast imaging became possible only with the advancement of fast microscopy techniques, e.g., resonant scanners or spinning disc devices. In contrast to measurements of cSOCE, where SR Ca^2+^ levels are strongly decreased, pSOCE was activated under conditions when SR Ca^2+^ stores were full, i.e., loaded to endogenous levels prior to activation. While these bulk SR Ca^2+^ levels are largely maintained upon low-frequency EFS [45,46,112], SOCE was found to be activated with individual APs nevertheless [39]. 

What were the main arguments for actually defining this Ca^2+^ influx as being store-dependent? First of all, the amount of Ca^2+^ lost in the t-system due to the activation of pSOCE showed a clear dependence on the amount of Ca^2+^ released from the SR upon an AP [39], which is the defining property of SOCE. If not SOCE, this dependence could also be explained by a Ca^2+^-dependent mechanism in which Ca^2+^ released from the SR per se would activate Ca^2+^-dependent ion channels at the sarcolemma, as it is known, e.g., from the family of transient receptor potential (TRP) channels. This, however, was ruled out by showing that pSOCE was unaffected when the fiber was bathed in a solution buffered with the fast Ca^2+^-chelating agent BAPTA instead of EGTA [39]. Second, pSOCE was blocked when Ca^2+^ release from the SR was inhibited by blocking the RyR1. Thus, micromolar concentrations of both tetracaine and ryanodine abolished SR Ca^2+^ release and pSOCE [39]. Moreover, increasing [Mg^2+^]_cyto_ from 1 to 3 mM, a well-known condition that inhibits RyR activity, largely suppressed SR Ca^2+^ release, in agreement with previous findings [107,113,114], and abolished pSOCE [39]. Of note, while tetracaine and ryanodine can affect Ca_v_1.1 and Na_v_1.4 function, despite using higher concentrations than reported within this study [39], increased [Mg^2+^]_cyto_ did not affect t-system excitability [113,114]. Third, pSOCE was reduced by increasing the SR Ca^2+^ load. Thus, exposing the fiber to [Ca^2+^]_cyto_ that was increased from an approximately physiological level of 67 nM to 200 nM and 1.3 µM led to an increase in [Ca^2+^]_SR_ and a subsequent reduction in pSOCE [111], proposedly by increasing the distance (concentration-wise) to the threshold of SOCE activation. The observed inhibition was not due to the increased [Ca^2+^]_cyto_ *per se*, as preloading the fiber at an increased [Ca^2+^]_cyto_ of 200 nM and then returning it to the original [Ca^2+^]_cyto_ of 67 nM immediately before the pSOCE assessment (establishing identical recording conditions but different SR Ca^2+^ load) resulted in marked suppression of pSOCE at the beginning of the recording, again in agreement with an increased distance to the threshold of SOCE activation.

### 3.3. A Potential Model of Phasic SOCE Activation 

**Figure 4:**
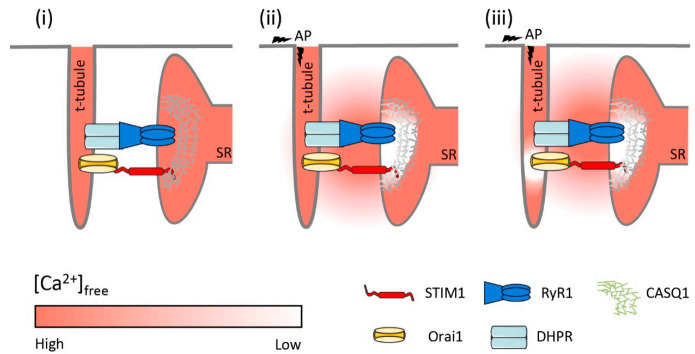
Proposed model of pSOCE activation following AP-evoked Ca^2+^ release in skeletal muscle. (**i**) At rest, some STIM1–Orai1 complexes are prearranged at the junctional membranes close to the position of the RyRs. (**ii**) During AP-evoked Ca^2+^ release from the SR, Ca^2+^ depletion within the SR occurs locally and is restricted to a nanodomain behind the RyR channels, which are presumably shaped by the network of CASQ. (**iii**) The local depletion of Ca^2+^ in a spatially restricted domain behind the RyRs allows for rapid dissociation of Ca^2+^ from STIM1 to activate SOCE.

pSOCE was activated with every AP under conditions where the bulk SR Ca^2+^ levels were barely depleted [45,46,112]. How is this possible? A potential model to explain pSOCE activation despite full SR Ca^2+^ stores was recently proposed by Koenig et al. [39,111]; it is shown in Figure 4 and discussed briefly. 

Under resting conditions, free [Ca^2+^]_SR_ was reported at about 0.5 mM in intact FDB fibers [115] and 0.31 mM in mouse *tibialis anterior* (TA) muscle [112]. In rat skinned EDL fibers with [Ca^2+^]_cyto_ set to 67 nM, free [Ca^2+^]_SR_ was determined to be 0.7 mM [111]. The luminal Ca^2+^-affinity of STIM1 was estimated to be around 0.2 mM, with reported Kd_Ca_ values of STIM1 between 200 and 600 µM [116], ~200 µM [117], ~150 µM [26], and an estimated upper limit of 300 µM [111]. Thus, under resting conditions when [Ca^2+^]_SR_ is between 0.5–0.7 mM, STIM1 luminal Ca^2+^-binding sites must be mostly occupied; bulk [Ca^2+^]_SR_ does not drop significantly during an AP [45,46,112] and, hence, cannot be critical for pSOCE activation. Steady [Ca^2+^]_SR_ is maintained by the strong buffering of CASQ, which is the main Ca^2+^-buffer in the SR [118,119,120]. CASQ is essentially absent in the lSR [121] but is concentrated at the jSR, where it provides large amounts of Ca^2+^ to be released despite maintaining [Ca^2+^]_SR_ at a steady level. This is accomplished by the high buffering capacity of CASQ, which is further increased upon polymerization [122]. When [Ca^2+^]_SR_ is high, CASQ forms long polymeric tendrils that are anchored to the RyR1 via triadin and junctin [91,123]. These tree-like structures, also known as Ca^2+^ wires, are thought to facilitate the diffusion of Ca^2+^ toward the RyR1 channel mouth due to a reduction in dimensionality [124,125]. The structural inhomogeneity of Ca^2+^-buffer across the SR and the local release of Ca^2+^ through the RyR1 at the jSR must result in spatial and temporal differences in [Ca^2+^]_SR_ [45]. These SR Ca^2+^ gradients may be expected to be small when considering basal RyR1 leakage [90,126,127] but must become significant during AP-induced SR Ca^2+^ release. In particular, within the jSR, Ca^2+^ nanodomains will form in which Ca^2+^ concentrations will differ substantially from the Ca^2+^ concentrations present in the bulk SR; however, direct assessment of these concentrations was not possible in the past due to a lack of respective experimental means. Thus, transient changes in [Ca^2+^]_SR_ during EC coupling have been limited to global averages of bulk [Ca^2+^]_SR_ [45,46,112]. Recent advances have, however, overcome these limitations owing to the development of genetically encoded probes. Evidence for local Ca^2+^ microdomains was provided recently using a G-CatchER^+^ sensor fused to JP-45 [128] in mouse fast-twitch FDB fibers. Because JP-45 is an integral component of the triad that interacts with Ca_v_1.1 at its N-terminus and with CASQ at its C-terminal, it is localized to the jSR within the triad region. The G-CatchER^+^ Ca^2+^ probe fused to the C-terminal end can thus report the Ca^2+^ concentration within the lumen of the jSR. While this Ca^2+^ probe was too slow to catch changes associated with single muscle twitches, it nevertheless proved an important principle: Ca^2+^ levels dropped much faster and deeper at the jSR when compared to the bulk SR. This was shown for fibers that were activated with tetanic stimulation, but these experiments certainly imply that changes occur upon single muscle twitches also. It is therefore comprehensible from a physiological point of view that respective STIM1 Ca^2+^-sensing proteins are located at these sites [29,38,41] in close proximity to the RyR1, where they would react most sensitive to changes in free Ca^2+^. Other pools of STIM1 distributed along the lSR would sense bulk [Ca^2+^]_SR_ and hence react with much less sensitivity. Their role is likely not an immediate one, but may involve recruitment of additional Ca^2+^ entry pathways upon heavy demand or functional impairment. 

While cSOCE was attributed to STIM/Orai [38,89,129], the molecular correlates of pSOCE have not been elucidated to date. A simple model would assume that pSOCE is carried by the same protein machinery as chronic SOCE [39,111], but this has not been shown to date. Besides a coupling of STIM1 with Orai1, two other main alternatives come to mind considering current knowledge: (i) Ca^2+^-channels other than Orai1, e.g., from the TRP family of ion channels, could mediate pSOCE, although some TRP channels have been neglected as mediators of cSOCE. For example, cSOCE was shown to be unaffected by the knockdown of TRPC3 [130] or by transient expression of dominant-negative TRPC3 and TRPC6 [131]. While apparently not mediating cSOCE, they could potentially carry pSOCE, either alone or together with Orai1 within a heteromeric channel structure [132]. Thus, the coupling of TRPC1 and TRPC4 to STIM1L was recently suggested to underlie fast SOCE in muscle [133], and a ternary complex of TRPC1, Orai1, and STIM1 was proposed to underlie SOCE in human salivary glands and platelets [132,134,135,136]. (ii) It is also possible that Orai is not gated by STIM proteins but by direct conformational coupling to RyR1 [131,137]. Thus, Orai1 could couple to RyR1 in a similar way, as it was shown for TRPC channels and RyR1 [138,139]; coupling to the RyR1 would confer the necessary “store-dependence,” not by sensing SR luminal Ca^2+^, but by coupling Orai1 activation to the conformational changes in RyR1. Apart from RyR1, it was also suggested that cSOCE involves coupling to IP_3_R [94,140], although IP_3_R expression in skeletal muscle is relatively low and preferentially confined to the nuclear envelope and NMJ [52,141].

Elucidating the molecular correlates of pSOCE was limited by non-selective pharmacology (see below) and the fact that pSOCE measurements originally described in rat EDL muscle fibers [39,111] were not easily translatable to mouse muscle fibers. Thus, EFS worked well in rat EDL fibers but, for reasons still unknown, mouse EDL fibers were found to be much less susceptible to EFS. Only recently, Lilliu et al. managed to overcome this limitation by training mice before SOCE measurements were performed [142]. Thus, providing running wheels to the cages of otherwise physically inactive mice enabled successful EFS in mouse skinned EDL fibers. Access to the running wheels for 5–6 days proved sufficient for EFS to work in mouse fibers in a manner that was completely comparable to rat fibers. pSOCE assessed in EDL fibers from these trained mice was indistinguishable from that in rat, suggesting the presence of pSOCE across mammalian species. Importantly, this new experimental paradigm opened the door to use genetically modified mice to test for the molecular nature of pSOCE.

### 3.4. What Is the Physiological Role of Phasic SOCE?

The demonstration of pSOCE upon individual APs [39,111,142] shed new light on the physiological role of SOCE in skeletal muscle function in general. Given that pSOCE was activated already with the first AP under conditions of endogenous SR Ca^2+^ loads suggested a primary physiological role of pSOCE and potentially of cSOCE, independent of SR Ca^2+^ store refilling. Of note, pSOCE was best assessed under conditions of high cytosolic buffering (10 mM EGTA; [39]). The high EGTA strongly buffered the [Ca^2+^]_cyto_ that was released from the SR to keep [Ca^2+^]_cyto_ at relatively low levels during EC coupling. This ensured a larger Ca^2+^ gradient across the t-system membrane and hence enabled a larger pSOCE flux (increased driving force compared to low EGTA buffering conditions). When EGTA was lowered to 0.2 mM, equivalent to the endogenous Ca^2+^-buffering power in fast-twitch muscle [39,143], pSOCE could not be resolved anymore [39], indicating no net Ca^2+^ flux under these conditions. Apparently, [Ca^2+^]_cyto_, in particular within the triadic cleft, reaches levels equaling that within the t-system during a twitch such that pSOCE operated near the reversal potential of Orai with a strongly reduced driving force (the depolarized membrane potential of the AP will not greatly affect the driving force for pSOCE as the AP is almost fully depolarized before Ca^2+^ reaches its peak in the cytoplasm (e.g., [144,145]), and pSOCE was activated only an additional 0.3 ms after SR Ca^2+^ release [111]). 

Another explanation for the decline in pSOCE amplitude with low cytosolic buffering could be provided by the reported inward rectification of the current–voltage relationship of Orai1 [24]. While this seems to be an intrinsic property of Orai1 channels rather than being caused by the asymmetric ionic composition of electrophysiological test solutions [24], it remains to be tested whether this rectification persists under near symmetric Ca^2+^ concentrations at a high micromolar [Ca^2+^]_cyto_, as is the case during a twitch. Irrespective of the biophysical mechanism behind the phenomenon, the data suggests that pSOCE, in cooperation with the t-system Ca^2+^-uptake proteins Na^+^/Ca^2+^ exchanger (NCX) and/or plasma membrane Ca^2+^ ATPase (PMCA), equilibrates Ca^2+^ gradients across the t-system membrane in a bidirectional manner, leading to an influx of Ca^2+^ if [Ca^2+^]_cyto_ transients are lower and an efflux of Ca^2+^ if [Ca^2+^]_cyto_ transients are higher than “normal.” These considerations suggest that pSOCE regulates fiber Ca^2+^ on a twitch-to-twitch basis by correcting changes in SR Ca^2+^ release. Thus, rather than refilling depleted SR Ca^2+^ stores, the role of pSOCE is probably better described as maintaining SR Ca^2+^ levels. One could also see pSOCE as a mechanism that prevents excessive build-up of Ca^2+^ within the t-system when [Ca^2+^]_cyto_ levels peak during a muscle twitch. By “clamping” the t-system Ca^2+^ levels to the levels found in the extracellular space, the function of pSOCE would therefore avoid significant diffusional loss of Ca^2+^ from the t-system into the external environment of the fiber [39,111].

As mentioned previously, it remains to be confirmed whether pSOCE depends on the very same protein machinery as cSOCE, i.e., STIM and Orai proteins, but it is also possible that their physiological function and localization are different. Based on what is known today, one can postulate (at least) two pools of SOCE to exist in skeletal muscle cells: one endogenous pool that can be activated rapidly and most likely relies on preformed clusters within the triads (see above); the other one, an inducible pool, is recruited only slowly after strong SR Ca^2+^ store depletion and might involve additional membrane structures [42,85], potential relocation of STIM proteins [29,146], and recruitment of additional Orai1 channels [42]. It is possible that the inducible pool of SOCE represents a condition of protein “overexpression,” which arises only under unphysiological conditions of SERCA block-induced store depletion, where an inducible pool of SOCE will outperform endogenous SOCE. Thus, its physiological relevance in skeletal muscle should be considered with caution.

## 4. Pharmacology of SOCE in Skeletal Muscle: Recent Advances

Since even before the molecular mechanisms of SOCE were elucidated, drugs from the group of imidazoles (e.g., SKF-96365; [147,148]), diphenylboronate (e.g., 2-APB; [149]), and pyrazoles (e.g., BTPs; [150,151,152]; further discussed below) were used to target SOCE. With the discovery of STIM and Orai, SOCE pharmacology significantly advanced based on the recombinant expression of respective proteins in non-excitable cells. However, data on the effects of the most commonly used SOCE modulators in muscle cells and, specifically, in skeletal muscle fibers, are sparse. In this section, we therefore give an overview of the current knowledge on SOCE pharmacology in skeletal muscle, with a particular focus on the common practice and recent developments in the field. For an overview of general SOCE pharmacology, the reader is referred to excellent reviews elsewhere (e.g., [24,153,154,155]).

Lanthanides (Gd^3+^ and La^3+^) were among the first modulators ever to target Orai1. They bind Orai1 and its isoforms Orai2 and Orai3 with high potency by blocking the pore [156]. Both La^3+^ and Gd^3+^ were widely used in skeletal muscle as standard SOCE inhibitors at micromolar concentrations (1–200 µM; [38,157,158,159,160]). While the affinities of SOCE and the different Orai isoforms for lanthanides lie in the nM range [24], their usage in the (high) micromolar range is common practice in the skeletal muscle field (e.g., [29,38,159]). At these concentrations, Gd^3+^ and La^3+^ also bind voltage-gated Ca^2+^ and TRP channels [161,162]. Indeed, lanthanides were also used as inhibitors of excitation- coupled Ca^2+^ entry (ECCE; e.g., [163,164,165]), which is most probably mediated by Ca_v_1.1 [166]. 

SKF96365 was first described as a receptor-mediated Ca^2+^ influx blocker [147] and only later as a SOCE blocker in Jurkat T cells [167]. Studies performed in non-excitable and skeletal muscle cells highlighted its low selectivity, as it targets a wide variety of ion channels, including voltage-gated Ca^2+^ [96,148,166,168], TRPM4 [169], and K_ATP_ channels [170]. Moreover, SKF96365 at concentrations in the micromolar range was shown to affect ER Ca^2+^ pumps in human endothelial cells [171] and NCX in glioblastoma cells with an EC_50_ of ~10 µM [172]. To block SOCE in skeletal muscle, SKF96365 is commonly applied at a concentration of 20–30 µM [38,59]. Within the same concentration range, it is used to block ECCE [166,173]; it also blocked APACC at 25 µM [96]. Taken together, these studies show that SKF96365 inhibits extracellular Ca^2+^ entry into skeletal myotubes and muscle fibers mediated by SOCE, ECCE, or APACC with similar potency.

2-Aminoethyldiphenyl borate (2-APB) was widely used to modulate Ca^2+^ fluxes and I_CRAC_ in a variety of tissues and cell types, including skeletal muscle [149,157,174,175]. It has a complex pharmacological profile in terms of both selectivity and mechanism of action. Initially, it was described as an IP_3_R antagonist [51,149,176,177,178], only to be later recognized as an SOCE blocker [179,180,181]. More recent findings in non-excitable cells have also highlighted the ability of 2-APB to modulate STIM1 multimerization and/or STIM1–Orai1 interaction [182,183], as well as the activity of potassium channels, SERCA pumps, TRPV1-2-3 channels, and interestingly, the DHPR [166,168]. Interestingly, 2-APB also shows a bi-phasic effect by which it can potentiate I_CRAC_ at low concentrations (1–10 µM), while higher concentrations (20–50 µM) inhibit I_CRAC_ [180,182,184]. Such a feature also affected the modulation of individual Orai isoforms. In HEK cells, 5 µM of 2-APB potentiated currents through Orai1 and Orai2 with no effect on Orai3, while 50 µM strongly inhibited Orai1 but potentiated Orai3-mediated currents [156,182,184]. Taken together, these observations suggest that 2-APB does not constitute a valuable tool when looking at specific channel activity.

3,5-bis(trifluorometyl)pyrazole (BTP) compounds were characterized for the first time as nuclear factor of activated T-cells (NFAT) activators and cytokine production inhibitors of T cells [150,151,152]. It was only later when BTP2 (also known as YM58483 or Pyr2) was described as a potent I_CRAC_ inhibitor [185,186,187]. During recent years, multiple studies reported the low selectivity of BTP2, as it was shown to potentiate the activity of TRPM4 [188] and to block TRPC3 and TRPC5 [189]. Despite its low selectivity, it is widely used as a SOCE inhibitor in skeletal muscle [38,41,87,110,190,191] at a concentration of 10 µM. Recently, a more detailed characterization of its effect in skeletal muscle fiber Ca^2+^ handling was provided [192]. The authors observed that BTP2 affects Ca^2+^ handling in rat skinned EDL fibers at multiple levels. It inhibited SOCE at low concentrations (1–5 µM) but, interestingly, also impaired RyR1 activity at higher concentrations. Thus, it blocked AP-induced SR Ca^2+^ release and RyR leak at >10 µM. The impairment of RyR1 occurred in an indirect manner, probably by affecting the DHPR [192], which is a well-known target of other SOCE modulators (e.g., 2-APB and SKF96365 [168]).

GSK-7975A and relatives (GSK-5503A, GSK-5498A) belong to the class of pyrazole derivatives developed by GlaxoSmithKline [193,194,195] that are widely used as I_CRAC_ inhibitors. GSK-7975A was shown to inhibit Orai1-mediated currents with an IC_50_ of ~4 µM in a heterologous expression system, presumably by altering the Orai1 pore geometry [194,196]. A recent work done in skeletal muscle showed the ability of this compound to block Mn^2+^ entry upon store depletion in myoblasts expressing TAM-associated Orai1 mutants, and thus suggested it as a potential candidate drug for TAM treatment [196]. Nevertheless, GSK-7975A does not exhibit high selectivity, as it also affects TRPV6 and L-type Ca^2+^ channels at 10 µM [194], and other Orai isoforms, i.e., Orai2 and, to a lesser extent, Orai3 [156]. The mechanism of SOCE inhibition by GSK-7975A appeared to be independent of STIM1–Orai1 coupling or STIM1-oligomerization, but was suggested to involve an allosteric modulation of the Orai1 selectivity filter [194]. However, the observed slow onset of inhibition (several minutes) argues against such a direct effect on the channel pore [24]. To the best of our knowledge, GSK compounds have not been applied to myotubes or skeletal muscle fibers such that respective insights are lacking to date.

Synta66 is structurally closely related to BTP2 and inhibits Orai1 activity at micromolar concentrations via a pore-blocking mechanism [197]. It has weaker reported off-target effects on other ion channels including TRPC1/5 [198], voltage-gated Ca^2+^ and Na^+^ channels [199], and other Orai isoforms [156]. However, comparable to BTP2 and the GSK compounds, the speed of inhibition by Synta 66 is rather slow such that cells need to be preincubated for more than 1 h for proper block development [24]. Together with the reported poor reversibility of drug action, this severely hinders the application of Synta66 in muscle fiber experiments. A more comprehensive characterization of the effects of Synta66 and its newly developed derivative in skeletal muscle is currently lacking.

### Recent Drug Developments and Future Perspectives

Recent attempts have led to the synthesis of novel SOCE inhibitors, of which CIC-37 seems the most promising candidate [200], but also to the discovery of SOCE enhancers, e.g., AL-2T, NM-3G [200], and “compound 47” [201]. However, a respective evaluation of their effects on SOCE in skeletal muscle is to date pending. 4-((5-phenyl-4-(trifluoromethyl)-thiazol-2-yl)amino)benzoic acid (IA65) is currently the only compound amongst the few reported SOCE enhancers whose effects have been characterized to some extent. This was done not only in heterologous expression systems but also in vascular smooth muscle cells and skeletal muscle fibers [202]. Here, IA65 increased the Orai1-mediated current and accelerated CDI in a concentration-dependent manner with an EC_50_ of 2 µM. Ca^2+^ influx through SOCE measured in rat skinned EDL fibers upon exposure to caffeine and low Mg^2+^ showed a comparable increase by about 20–30% at 10 µM IA65. IA65 exhibited some selectivity, enhancing Orai1 activity but only marginal affecting Orai2 and Orai3 [156,202]. It remains to be seen whether these novel drugs will provide helpful tools to study SOCE in skeletal muscle.

What can be learned from the existing data? While drug specificity is always of concern when applying pharmacology, it seems that this particularly applies to SOCE: (i) the different Ca^2+^ influx pathways across the t-tubule membrane are difficult to discriminate with current pharmacology; (ii) the importance of several proteins to the function of SOCE, be it direct (STIM, Orai) or indirect (RyR, SERCA, etc.), greatly increase the probability for off-target effects; (iii) the RyR plays a pivotal role in SOCE activation [30,39,59,89,97] but is characterized by substantial drug binding promiscuity toward small molecules and complex drug–receptor interactions, as exemplified by some of the better-studied molecules, namely, ryanodine and tetracaine (rev in [203]). In particular, the situation becomes delicate when considering fully mature muscle fibers, when all of the above applies, and interpretations solely based on drug action are not straightforward, as demonstrated, e.g., by Meizoso-Huesca [192]. Regarding pSOCE, another level of complexity is added when the whole EC-coupling machinery may constitute a potential drug target, including Ca_v_1.1 and Na_v_1.4, which are known to be affected by current SOCE pharmacology. 

## 5. Variety of STIM Molecules Expressed in Adult Tissue

Skeletal muscle does not only express STIM1 but also a long splice variant, namely, STIM1L, as well as two STIM2 isoforms. STIM1L, which results from the alternative splicing of the STIM1 gene at exon 11, was discovered 10 years ago to be expressed in human myotubes [146]. In contrast to STIM1, STIM1L is not ubiquitously expressed but is restricted to skeletal and cardiac muscle, as well as to the brain, at least in mice. Another study reported STIM1L mRNA to be exclusively expressed in human skeletal muscle [204]. STIM1L has an extra 106 amino acids in the C-terminal region, which comprises a putative actin-binding domain. STIM1L is absent in myoblasts, and its expression increases during differentiation, eventually reaching a similar level as STIM1 in adult tissue [146]. STIM1L was shown to be pre-localized at the PM before store depletion in clusters co-localizing with Orai1. Knockdown of STIM1L specifically (but not STIM1) slowed down the activation kinetics of SOCE [146], pointing to a specific function of STIM1L in the fast activation of skeletal muscle SOCE. It should be stressed that these results were obtained from experiments that were performed on differentiating myotubes before triad formation had occurred; thus, the localization of STIM1L in adult fibers remains to be determined. Subsequent studies on the intrinsic properties of STIM1L revealed that when expressed in non-muscle cells (mouse embryonic fibroblasts double KO for STIM1/2, MEFDKO), STIM1L did not lead to a faster activation of SOCE, even if the protein was pre-localized at the PM prior to store depletion [205]. This could be explained by the fact that Orai1 did not form clusters co-localizing with STIM1L in MEFDKO before store depletion, as seen in myotubes. These data further imply that additional proteins are necessary for the formation of permanent “preassembled” clusters of STIM1L and Orai1 in myotubes. Additional experiments will tell whether STIM1L is indeed necessary and sufficient for fast SOCE in skeletal muscle. Using MEFDKO cells, it was further shown that SOCE was larger when induced by STIM1L compared to STIM1 [205]. Interestingly, however, the cER did not enlarge after store depletion, as is the case in STIM1 overexpressing cells [17]. Hence, the higher SOCE took place even at reduced contact between the ER and the PM. Several hypotheses can potentially explain the enhanced Ca^2+^ entry induced by STIM1L. A reduction in ER-PM contacts could lead to less Ca^2+^ accumulation in the proximity of the Orai1 channel to reduce CDI and hence augment SOCE. Another explanation is linked to different channels that are activated by STIM1L. Electrophysiological recordings in HEK cells expressing STIM1 or STIM1L showed that STIM1L is indeed more prone to activating TRPC1 compared to STIM1 [206]. On the contrary, STIM1 is a better Orai1 opener than STIM1L, but in HEK, as well as in MEF cells, STIM1L-induced SOCE was larger than STIM1-induced SOCE [206]. Along this line, Horinouchi et al. [204] reported that STIM1L induced more Ca^2+^ entry than STIM1 and interacts more strongly with TRPC (in that case TRPC3 and TRPC6) than STIM1. Interestingly, in differentiated human myotubes, it was shown that STIM1L indeed physically interacts with TRPC1 and TRPC4 [133]. Furthermore, the downregulation of either STIM1L or TRPC1/4 resulted in similar phenotypes, such as a reduced myoblast fusion into myotubes and an impaired ability to sustain repetitive SR Ca^2+^ release [133,207]. Along this line, the kinetics of SOCE was slower in myotubes with reduced STIM1L or TRPC1/4 expression [133]. Hence, it appears that besides Orai1, TRPC channels are involved in muscle SOCE, in particular, the ones activated by STIM1L, and that this plays an important role for myotube formation and their ability to release Ca^2+^ (rev in [208]).

The other member of the STIM family, namely, STIM2, is expressed in muscle as well and plays a role in differentiation. The downregulation of STIM2 in human skeletal muscle led to an impaired myoblast differentiation, a decreased expression of myogenin and myocyte enhancer factor-2 (MEF2), and a reduction in SOCE [209]. In addition, the basal cytosolic Ca^2+^ level was reduced both in myoblasts and myotubes, in line with several reports showing the role of STIM2 in the maintenance of resting Ca^2+^ levels in different cell types [210]. Interestingly, the level of STIM2 expression increased during the process of muscle differentiation, which could point to an important role in adult tissue as well. However, STIM2 KO mice did not display alterations in muscle function, which might be explained by an overall low expression level of STIM2 in mice [211]. Considering the strong effect of siSTIM2 on SOCE in human myotubes (about an 80% reduction; [209]), one can speculate that human skeletal muscle expresses STIM2 at a higher level than mouse muscle, or that STIM2 is more important for SOCE in human tissue. Indeed, the downregulation of STIM2 reduced SOCE by only 20% in mouse myotubes [212]. Besides the effect of STIM2 on SOCE, SERCA1a activity was enhanced in STIM2-downregulated cells [212]. Accordingly, the SR Ca^2+^ content was higher and, consequently, KCl- and caffeine-induced Ca^2+^ release was more pronounced in the case of STIM2 downregulation. The cytosolic part of STIM2 was identified as the binding region to SERCA1a [212]. In contrast, in human myotubes, KCl-induced Ca^2+^ release was reduced in STIM2-downregulated cells and repeated KCl stimulations rapidly led to SR Ca^2+^ store depletion [209]. The conflicting results obtained after the downregulation of STIM2 might have been due to species differences or the presence of an alternative STIM2 splice form. In 2015, two groups discovered an inhibitory isoform of STIM2, called STIM2β or STIM2.1, which has an insertion of eight amino acids in the CAD domain of STIM2, preventing Orai1 gating; the other STIM2 was called STIM2a or STIM2.2. [213,214]. Interestingly, the expression of this splice form increased in differentiating myotubes [213,215]. The KO of STIM2.1 in C2C12 muscle cells showed a decreased differentiation process, as highlighted by a reduced expression of myosin heavy chain and myogenin [215]. Furthermore, MEF2C and NFAT4 expression were reduced, suggesting that an increased expression of STIM2.1 during myogenesis promotes the formation of myotubes through MEF2C and NFAT4. In line with the inhibitory role of STIM2.1, SOCE was slightly elevated in cells lacking this isoform. Finally, myoblast proliferation was increased in cells lacking STIM2.1, possibly due to the basal cytosolic Ca^2+^ level elevation that was observed under this condition [215]. This effect on the basal Ca^2+^ level was, however, not seen in CD4^+^ T cells [214], which might point to different functions of STIM2.1 depending on the specific cell type. What should also be considered is the ratio between both STIM2 isoforms (STIM2.1/STIM2.2), which is likely different between T cells and muscle. In addition, this ratio changes during myogenesis, with an increased expression of the inhibitory STIM2.1 isoform. This might help to prevent excessive Ca^2+^ entry during muscle formation, considering that STIM1L expression also increases during muscle differentiation and promotes SOCE [146]. It is well known that overactive SOCE is detrimental for muscle formation/function, as evidenced by the gain-of-function mutation in either STIM1 or Orai1 that leads to TAM [216]. Hence, the expression of an inhibitory isoform, such as STIM2.1, during myogenesis likely participates in the tight regulation of Ca^2+^ homeostasis during muscle formation, but also in mature fibers. We are now only at the beginning of understanding the complex regulation of SOCE that is linked to the expression of STIM proteins, and future studies should bring exciting new findings to better understand the interplay between the four STIM isoforms that are expressed in skeletal muscle.

## 6. STIM and Orai in Different Fiber Types

Based on the different properties regarding the speed of contraction, metabolism, and fatigue resistance, different types of muscle fibers can be distinguished in skeletal muscle. According to their contraction velocity, two main types are defined, namely, type I and type II, which are also called slow- and fast-twitch fibers, respectively [217]. The different expression of myosin heavy chain (MHC) isoforms and ATPase activity (rev in [218]) allows for further subdividing adult fibers into four groups, from the slowest one to the fastest one (IIB < IIX < IIA < I, with IIB not being found in humans). In fast-twitch fibers, the Ca^2+^ release upon an AP has a higher amplitude and shorter duration than in slow-twitch fibers [219]. The faster muscle relaxation speed can be explained by a better organization of the SR and t-tubules in the fast-twitch fibers [220] and a more robust expression of the RyR1 and SERCA proteins [219]. Skeletal muscle fiber composition is heterogeneous depending on the muscle type, and muscles containing more slow-twitch fibers, such as in soleus muscles, resist fatigue better and are mainly responsible for the maintenance of posture. In contrast, muscles that are mainly composed of fast-twitch fibers, such as the EDL, are more sensitive to fatigue and are mainly used for intensive and short-time contractions.

The contribution of SOCE toward maintaining contractile force during repetitive muscle activity in the different fiber types is not clearly defined for the time being. Ex vivo experiments showed that the contractile force of soleus muscles, upon stimulation at high frequencies, is more dependent on extracellular Ca^2+^ than that of EDL muscles. Hence, in soleus muscles, the force of contraction decreased more strongly in the presence of BTP2 or the absence of extracellular Ca^2+^ [190], showing a greater implication of SOCE to avoid fatigue during intensive muscle contractions in slow-twitch fibers. Still, as SOCE inhibition affected both fiber types during tiring stimulations, it is likely that the role of SOCE is the same in the two types of fibers. However, the relative importance of SOCE for preventing muscle fatigue is more pronounced in muscles that are more frequently used, as is the case for slow muscle. Interestingly, upon low-frequency stimulation, SOCE inhibitors did not enhance fatigue, neither in slow- nor in fast-twitch muscle.

A critical role of SOCE in slow muscle was demonstrated recently in muscle-specific Orai1 knock-out mice [129]. The reduction in specific force in soleus muscle from Orai1 KO animals was stronger than in EDL muscle. In addition, Orai1 deletion more markedly affected soleus muscle with a reduction in the percentage and the cross-section area of slow-twitch fibers (type I) in adult mice. As the reduction in muscle force was not observed in inducible Orai1 KO animals (where Orai1 expression was reduced only at adulthood), it was concluded that the decreased specific force specifically in soleus muscle was due to a developmental defect rather than a lack of Ca^2+^ entry during EC coupling [129]. It should also be noted that in human patients, Orai1 loss of function, which in this case is not muscle-specific, induces atrophy of fast fibers, with a resulting predominance of slow fibers ([221] and rev in [86]). Why slow and fast fibers are differently affected by the absence/loss of function of Orai1 is not clear. It could be related to species variants, i.e., human versus mouse, or differences in the tissues lacking Orai1, i.e., all cells versus skeletal muscle. More investigations are required to firmly establish the function of Orai1 (and SOCE) across fiber types.

Another interesting point is related to the formation of tubular aggregates (TAs), which are abnormal structures of the SR that strongly express STIM1 and Orai1 [222]. In humans, TAs are described in patients with a gain-of-function mutation of Orai1 or STIM1 (rev in [216]). It was proposed that these very compact SR structures sequester STIM1 and Orai1 to protect the muscle cell from excess Ca^2+^. In mice, TAs are found in old animals almost exclusively in type IIB fibers [223], which are the fastest, most easily tired ones, but also the least used fibers (rev in [224]). Interestingly, when older mice were allowed to run in a wheel during most of their life span (between 9 and 24 months), the formation of TAs was reduced [222]. In this case, the old muscles from trained mice required extracellular Ca^2+^ entry for a stronger contraction and are sensitive to SOCE inhibitors [222], which was not the case in old, untrained animals [190]. Indeed, as mentioned previously (Section 2.2), it was proposed that in old animals, SOCE is not required to maintain force, essentially because SOCE is much less functional with age [73]. The decline in SOCE function with age is, however, in contradiction with another study reporting fully functional SOCE in old animals [76]. From these animal studies, one can hypothesize that the more muscles are used, especially slow muscles, the more SOCE is needed to fight fatigue during intense effort. In very fast fibers, such as IIB fibers, muscle usage overall is less frequent and even less during aging, and the role of SOCE to prevent fatigue is less pronounced. However, the fast fibers develop TAs with age, possibly as a protective mechanism against Ca^2+^ overload. Slow fibers, with a higher mitochondrial density and a preferential oxidative metabolism, might be better protected against Ca^2+^ overload, thus not requiring TAs formation, but this remains hypothetical. As reported in a recent comprehensive review on CEUs, the structural rearrangement that occurs upon strenuous exercise was so far only studied/observed in fast muscle [84]. It would be of interest to know whether this is specific to fast fibers or whether slow fibers also undergo such t-tubule rearrangement. Considering the significant difference in Ca^2+^ handling between the two fiber types, it would not be surprising that SOCE is also divergent in terms of localization within the fiber and regarding respective activation/regulatory mechanisms.

Finally, what do we know about the expression of the SOCE molecules in the different fiber types? Not much is known besides a study that reported a higher expression level of STIM1L in rat soleus muscle compared to rat EDL muscle [97]. The level of STIM1 was, however, similar between slow and fast muscle [97]. Recently, two studies investigated the RNA expression in all nuclei of different mouse muscle cells using single nuclei-RNA seq [225,226]. When we re-analyzed the dataset, we found that in adults, the expression of STIM1 and STIM2 is not homogeneous in the different types of fibers. STIM1 is strongly expressed in type I, IIA, and IIX fibers in the adult soleus, while STIM2 is hardly detected in the soleus muscle. Surprisingly, in adult TA muscle, which is mainly made up of fast fibers, STIM1 is expressed more in IIX fibers than in faster IIB fibers, while conversely, STIM2 is expressed mainly in IIB fibers. Hence, to schematize, STIM1 is more a slow fiber protein, while STIM2 is a fast fiber one. These results highlight potentially distinct functions of STIM1 and STIM2 in different muscle fibers and might confirm a greater role of STIM1-induced SOCE in slow fibers. In contrast, the expression of Orai1 is more uniform in the different fibers of TA muscle [225,226]. The physiological consequences of such distinct expressions of STIM1 and STIM2 (assuming the protein expression follows the same pattern as the mRNA) open new exciting ways for further investigations to understand the function of both proteins and their splicing isoforms in skeletal muscle in depth.

## 7. Concluding Remarks

Skeletal muscle is not a homogenous tissue but comprises different types of fibers that have an extremely broad range of activity. The physiological function(s) and regulation(s) of SOCE in this context are so far only poorly understood. Several basic questions remain open, including the localization, mobility, and interaction of the involved SOCE molecules, as well as their differential expression among the different fiber types. Current methods for studying SOCE in muscle, some of which are specific to this tissue, such as the skinned fiber preparation and the force/fatigue measurements, have highlighted the peculiar and fascinating nature of this small but important Ca^2+^ flux. The picture of SOCE that emerges is a multifaceted one, envisaging SOCE as both static and dynamic in nature, where SOCE is not confined to the triad alone but acts presumably across multiple sites within a single fiber, where it likely serves different physiological functions. 

## Data Availability

All data presented in this review are present within the article or associated references.

## Abbreviations

AP	Action potential
APACC	AP-activated Ca^2+^ current
CAD	Ca^2+^-activated domain
CASQ	Calsequestrin
CDI	Ca^2+^-dependent inactivation
cER	Cortical ER
CEU	Ca^2+^ entry unit
CPA	Cyclopiasonic acid
CRACR2A	CRAC regulator 2A
DHPR	Dihydropyridine receptor
EB1	End-binding protein 1
EC	Excitation–contraction
EDL	Extensor digitorum longus muscle
EFS	Electrical field stimulation
FDB	Flexor digitorum brevis muscle
FRAP	Fluorescence recovery after photobleaching
HIT	High intensity
I_CRAC_	Ca^2+^ release activated Ca^2+^ current
IP_3_	Inositol 1,4,5-trisphosphate
IP_3_R	IP_3_ receptor
JPH	Junctophilin
jSR	Junctional SR
lSR	Longitudinal SR
MAP6	Microtubule-associated protein 6
MCS	Membrane contact site
MEF	Mouse embryonic fibroblast
MEF2	Myocyte enhancer factor-2
Mg29	Mitsugumin 29
MyHC	Myosin heavy chain
NCX	Na^+^/Ca^2+^ exchanger
NFAT	Nuclear factor of activated T-cells
NMJ	Neuromuscular junction
PiP_2_	Phosphatidylinositol 4,5-bisphosphate
PM	Plasma membrane
PMCA	Plasma membrane Ca^2+^ ATPase
POST	Partner of STIM
RyR1	Ryanodine receptor 1
SARAF	SOCE-associated regulatory factor
SERCA	Sarco-endoplasmic Ca^2+^ ATPase
SR	Sarcoplasmic reticulum
STIM	Stromal interaction molecule
STIMATE	STIM-activating enhancer
TA	Tibialis anterior muscle
TAs	Tubular aggregates
TC	Terminal cisternae
Tg	Thapsigargin
TRP	Transient receptor potential

## References

[1] Boncompagni S., Pozzer D., Viscomi C., Ferreiro A., Zito E. (2020). Physical and Functional Cross Talk Between Endo-Sarcoplasmic Reticulum and Mitochondria in Skeletal Muscle. Antioxid. Redox. Signal..

[2] Al-Qusairi L., Laporte J. (2011). T-Tubule Biogenesis and Triad Formation in Skeletal Muscle and Implication in Human Diseases. Skelet. Muscle.

[3] Sorrentino V. (2011). Sarcoplasmic Reticulum: Structural Determinants and Protein Dynamics. Int. J. Biochem. Cell Biol..

[4] Schiaffino S., Reggiani C. (2011). Fiber Types in Mammalian Skeletal Muscles. Physiol. Rev..

[5] Flucher B.E., Tuluc P. (2017). How and Why Are Calcium Currents Curtailed in the Skeletal Muscle Voltage-Gated Calcium Channels?: Curtailed Skeletal Muscle Calcium Currents. J. Physiol..

[6] Putney J.W. (1986). A Model for Receptor-Regulated Calcium Entry. Cell Calcium.

[7] Liou J., Kim M.L., Heo W.D., Jones J.T., Myers J.W., Ferrell J.E., Meyer T. (2005). STIM Is a Ca2+ Sensor Essential for Ca2+-Store-Depletion-Triggered Ca2+ Influx. Curr. Biol..

[8] Zhang S.L., Yu Y., Roos J., Kozak J.A., Deerinck T.J., Ellisman M.H., Stauderman K.A., Cahalan M.D. (2005). STIM1 Is a Ca2+ Sensor That Activates CRAC Channels and Migrates from the Ca2+ Store to the Plasma Membrane. Nature.

[9] Roos J., DiGregorio P.J., Yeromin A.V., Ohlsen K., Lioudyno M., Zhang S., Safrina O., Kozak J.A., Wagner S.L., Cahalan M.D. (2005). STIM1, an Essential and Conserved Component of Store-Operated Ca^2+^ Channel Function. J. Cell. Biol..

[10] Feske S., Gwack Y., Prakriya M., Srikanth S., Puppel S.-H., Tanasa B., Hogan P.G., Lewis R.S., Daly M., Rao A. (2006). A Mutation in Orai1 Causes Immune Deficiency by Abrogating CRAC Channel Function. Nature.

[11] Vig M., Peinelt C., Beck A., Koomoa D.L., Rabah D., Koblan-Huberson M., Kraft S., Turner H., Fleig A., Penner R. (2006). CRACM1 Is a Plasma Membrane Protein Essential for Store-Operated Ca^2+^ Entry. Science.

[12] Yeromin A.V., Zhang S.L., Jiang W., Yu Y., Safrina O., Cahalan M.D. (2006). Molecular Identification of the CRAC Channel by Altered Ion Selectivity in a Mutant of Orai. Nature.

[13] Huang G.N., Zeng W., Kim J.Y., Yuan J.P., Han L., Muallem S., Worley P.F. (2006). STIM1 Carboxyl-Terminus Activates Native SOC, Icrac and TRPC1 Channels. Nat. Cell. Biol..

[14] Liou J., Fivaz M., Inoue T., Meyer T. (2007). Live-Cell Imaging Reveals Sequential Oligomerization and Local Plasma Membrane Targeting of Stromal Interaction Molecule 1 after Ca^2+^ Store Depletion. Proc. Natl. Acad. Sci. USA.

[15] Park C.Y., Hoover P.J., Mullins F.M., Bachhawat P., Covington E.D., Raunser S., Walz T., Garcia K.C., Dolmetsch R.E., Lewis R.S. (2009). STIM1 Clusters and Activates CRAC Channels via Direct Binding of a Cytosolic Domain to Orai1. Cell.

[16] Lewis R.S. (2011). Store-Operated Calcium Channels: New Perspectives on Mechanism and Function. Cold Spring Harb. Perspect. Biol..

[17] Orci L., Ravazzola M., Le Coadic M., Shen W.-W., Demaurex N., Cosson P. (2009). STIM1-Induced Precortical and Cortical Subdomains of the Endoplasmic Reticulum. Proc. Natl. Acad. Sci. USA.

[18] Perni S., Dynes J.L., Yeromin A.V., Cahalan M.D., Franzini-Armstrong C. (2015). Nanoscale Patterning of STIM1 and Orai1 during Store-Operated Ca ^2+^ Entry. Proc. Natl. Acad. Sci. USA.

[19] Ercan E., Momburg F., Engel U., Temmerman K., Nickel W., Seedorf M. (2009). A Conserved, Lipid-Mediated Sorting Mechanism of Yeast Ist2 and Mammalian STIM Proteins to the Peripheral ER. Traffic.

[20] Bhardwaj R., Müller H.-M., Nickel W., Seedorf M. (2013). Oligomerization and Ca^2+^/Calmodulin Control Binding of the ER Ca2+-Sensors STIM1 and STIM2 to Plasma Membrane Lipids. Biosci. Rep..

[21] Berna-Erro A., Jardin I., Salido G.M., Rosado J.A. (2017). Role of STIM2 in Cell Function and Physiopathology: STIM2 in Cell Function and Physiopathology. J. Physiol..

[22] Muik M., Schindl R., Fahrner M., Romanin C. (2012). Ca^2+^ Release-Activated Ca^2+^ (CRAC) Current, Structure, and Function. Cell. Mol. Life. Sci..

[23] Cao X., Choi S., Maléth J.J., Park S., Ahuja M., Muallem S. (2015). The ER/PM Microdomain, PI(4,5)P2 and the Regulation of STIM1–Orai1 Channel Function. Cell Calcium.

[24] Prakriya M., Lewis R.S. (2015). Store-operated calcium channels. Physiol. Rev..

[25] Armstrong C.M., Bezanilla F.M., Horowicz P. (1972). Twitches in the Presence of Ethylene Glycol Bis(β-Aminoethyl Ether)-N,N′-Tetraacetic Acid. Biochim. Biophys. Acta (BBA)—Bioenerg..

[26] Launikonis B.S., Murphy R.M., Edwards J.N. (2010). Toward the Roles of Store-Operated Ca^2+^ Entry in Skeletal Muscle. Pflügers Arch.—Eur. J. Physiol..

[27] Ogawa Y., Kurebayashi N., Murayama T. (1999). Ryanodine Receptor Isoforms in Excitation-Contraction Coupling. Adv. Biophys..

[28] Murayama T., Kurebayashi N., Ogawa Y. (2000). Role of Mg(^2+^) in Ca(^2+^)-Induced Ca(^2+^) Release through Ryanodine Receptors of Frog Skeletal Muscle: Modulations by Adenine Nucleotides and Caffeine. Biophys. J..

[29] Stiber J., Hawkins A., Zhang Z.-S., Wang S., Burch J., Graham V., Ward C.C., Seth M., Finch E., Malouf N. (2008). STIM1 Signalling Controls Store-Operated Calcium Entry Required for Development and Contractile Function in Skeletal Muscle. Nat. Cell. Biol..

[30] Yarotskyy V., Dirksen R.T. (2012). Temperature and RyR1 Regulate the Activation Rate of Store-Operated Ca^2+^ Entry Current in Myotubes. Biophys. J..

[31] Mallouk N., Allard B. (2002). Ca(^2+^) Influx and Opening of Ca(^2+^)-Activated K(+) Channels in Muscle Fibers from Control and Mdx Mice. Biophys. J..

[32] Berbey C., Allard B. (2009). Electrically Silent Divalent Cation Entries in Resting and Active Voltage-Controlled Muscle Fibers. Biophys J..

[33] Kurebayashi N., Ogawa Y. (2001). Depletion of Ca^2+^ in the Sarcoplasmic Reticulum Stimulates Ca^2+^ Entry into Mouse Skeletal Muscle Fibres. J. Physiol..

[34] Lacruz R.S., Feske S. (2015). Diseases Caused by Mutations in *ORAI1* and *STIM1*: Mutations in *ORAI1* and *STIM1*. Ann. N. Y. Acad. Sci..

[35] Grigoriev I., Gouveia S.M., van der Vaart B., Demmers J., Smyth J.T., Honnappa S., Splinter D., Steinmetz M.O., Putney J.W., Hoogenraad C.C. (2008). STIM1 Is a MT-Plus-End-Tracking Protein Involved in Remodeling of the ER. Curr. Biol..

[36] Chang C.-L., Chen Y.-J., Quintanilla C.G., Hsieh T.-S., Liou J. (2018). EB1 Binding Restricts STIM1 Translocation to ER–PM Junctions and Regulates Store-Operated Ca^2+^ Entry. J. Cell. Biol..

[37] Barone V., Randazzo D., Del Re V., Sorrentino V., Rossi D. (2015). Organization of Junctional Sarcoplasmic Reticulum Proteins in Skeletal Muscle Fibers. J. Muscle. Res. Cell. Motil..

[38] Wei-LaPierre L., Carrell E.M., Boncompagni S., Protasi F., Dirksen R.T. (2013). Orai1-Dependent Calcium Entry Promotes Skeletal Muscle Growth and Limits Fatigue. Nat. Commun..

[39] Koenig X., Choi R.H., Launikonis B.S. (2018). Store-Operated Ca^2+^ Entry Is Activated by Every Action Potential in Skeletal Muscle. Commun. Biol..

[40] Lee K.J., Hyun C., Woo J.S., Park C.S., Kim D.H., Lee E.H. (2014). Stromal Interaction Molecule 1 (STIM1) Regulates Sarcoplasmic/Endoplasmic Reticulum Ca^2+^-ATPase 1a (SERCA1a) in Skeletal Muscle. Pflügers Arch.—Eur. J. Physiol..

[41] Boncompagni S., Michelucci A., Pietrangelo L., Dirksen R.T., Protasi F. (2017). Exercise-Dependent Formation of New Junctions That Promote STIM1-Orai1 Assembly in Skeletal Muscle. Sci. Rep..

[42] Sztretye M., Singlár Z., Balogh N., Kis G., Szentesi P., Angyal Á., Balatoni I., Csernoch L., Dienes B. (2020). The Role of Orai1 in Regulating Sarcoplasmic Calcium Release, Mitochondrial Morphology and Function in Myostatin Deficient Skeletal Muscle. Front. Physiol..

[43] Cusimano V., Pampinella F., Giacomello E., Sorrentino V. (2009). Assembly and Dynamics of Proteins of the Longitudinal and Junctional Sarcoplasmic Reticulum in Skeletal Muscle Cells. Proc. Natl. Acad. Sci. USA.

[44] Sébastien M., Aubin P., Brocard J., Brocard J., Marty I., Fauré J. (2020). Dynamics of Triadin, a Muscle-Specific Triad Protein, within Sarcoplasmic Reticulum Subdomains. MBoC.

[45] Launikonis B.S., Zhou J., Royer L., Shannon T.R., Brum G., Rios E. (2006). Depletion “Skraps” and Dynamic Buffering inside the Cellular Calcium Store. Proc. Natl. Acad. Sci. USA.

[46] Canato M., Scorzeto M., Giacomello M., Protasi F., Reggiani C., Stienen G.J.M. (2010). Massive Alterations of Sarcoplasmic Reticulum Free Calcium in Skeletal Muscle Fibers Lacking Calsequestrin Revealed by a Genetically Encoded Probe. Proc. Natl. Acad. Sci. USA.

[47] Blaauw B., del Piccolo P., Rodriguez L., Hernandez Gonzalez V.-H., Agatea L., Solagna F., Mammano F., Pozzan T., Schiaffino S. (2012). No Evidence for Inositol 1,4,5-Trisphosphate–Dependent Ca^2+^ Release in Isolated Fibers of Adult Mouse Skeletal Muscle. J. Gen. Physiol..

[48] Jaimovich E., Reyes R., Liberona J.L., Powell J.A. (2000). IP3 Receptors, IP3 Transients, and Nucleus-Associated Ca^2+^ Signals in Cultured Skeletal Muscle. Am. J. Physiol. Cell. Physiol..

[49] Casas M., Figueroa R., Jorquera G., Escobar M., Molgó J., Jaimovich E. (2010). IP3-Dependent, Post-Tetanic Calcium Transients Induced by Electrostimulation of Adult Skeletal Muscle Fibers. J. Gen. Physiol..

[50] Díaz-Vegas A.R., Cordova A., Valladares D., Llanos P., Hidalgo C., Gherardi G., De Stefani D., Mammucari C., Rizzuto R., Contreras-Ferrat A. (2018). Mitochondrial Calcium Increase Induced by RyR1 and IP3R Channel Activation After Membrane Depolarization Regulates Skeletal Muscle Metabolism. Front. Physiol..

[51] Powell J.A., Molgo J., Adams D.S., Colasante C., Williams A., Bohlen M., Jaimovich E. (2003). IP3 Receptors and Associated Ca^2+^ Signals Localize to Satellite Cells and to Components of the Neuromuscular Junction in Skeletal Muscle. J. Neurosci..

[52] Zhu H., Bhattacharyya B.J., Lin H., Gomez C.M. (2011). Skeletal Muscle IP3R1 Receptors Amplify Physiological and Pathological Synaptic Calcium Signals. J. Neurosci..

[53] Porter K.R., Palade G.E. (1957). Studies on the Endoplasmic Reticulum. III. Its Form and Distribution in Striated Muscle Cells. J. Biophys. Biochem. Cytol..

[54] Li C., Qian T., He R., Wan C., Liu Y., Yu H. (2021). Endoplasmic Reticulum–Plasma Membrane Contact Sites: Regulators, Mechanisms, and Physiological Functions. Front. Cell Dev. Biol..

[55] Hirata Y., Brotto M., Weisleder N., Chu Y., Lin P., Zhao X., Thornton A., Komazaki S., Takeshima H., Ma J. (2006). Uncoupling Store-Operated Ca^2+^ Entry and Altered Ca^2+^ Release from Sarcoplasmic Reticulum through Silencing of Junctophilin Genes. Biophys. J..

[56] van Oort R.J., Garbino A., Wang W., Dixit S.S., Landstrom A.P., Gaur N., De Almeida A.C., Skapura D.G., Rudy Y., Burns A.R. (2011). Disrupted Junctional Membrane Complexes and Hyperactive Ryanodine Receptors After Acute Junctophilin Knockdown in Mice. Circulation.

[57] Powell J.A., Petherbridge L., Flucher B.E. (1996). Formation of Triads without the Dihydropyridine Receptor Alpha Subunits in Cell Lines from Dysgenic Skeletal Muscle. J. Cell. Biol..

[58] Felder E., Protasi F., Hirsch R., Franzini-Armstrong C., Allen P.D. (2002). Morphology and Molecular Composition of Sarcoplasmic Reticulum Surface Junctions in the Absence of DHPR and RyR in Mouse Skeletal Muscle. Biophys. J..

[59] Pan Z., Yang D., Nagaraj R.Y., Nosek T.A., Nishi M., Takeshima H., Cheng H., Ma J. (2002). Dysfunction of Store-Operated Calcium Channel in Muscle Cells Lacking Mg29. Nat. Cell Biol..

[60] Thevenon D., Smida-Rezgui S., Chevessier F., Groh S., Henry-Berger J., Beatriz Romero N., Villaz M., DeWaard M., Marty I. (2003). Human Skeletal Muscle Triadin: Gene Organization and Cloning of the Major Isoform, Trisk 51. Biochem. Biophys. Res. Commun..

[61] Marty I. (2015). Triadin Regulation of the Ryanodine Receptor Complex: Triadin Regulation of the Ryanodine Receptor Complex. J. Physiol..

[62] Treves S., Vukcevic M., Maj M., Thurnheer R., Mosca B., Zorzato F. (2009). Minor Sarcoplasmic Reticulum Membrane Components That Modulate Excitation-Contraction Coupling in Striated Muscles: Sarcoplasmic Reticulum Membrane Components. J. Physiol..

[63] Wang Y., Li X., Duan H., Fulton T.R., Eu J.P., Meissner G. (2009). Altered Stored Calcium Release in Skeletal Myotubes Deficient of Triadin and Junctin. Cell Calcium.

[64] Treves S., Feriotto G., Moccagatta L., Gambari R., Zorzato F. (2000). Molecular Cloning, Expression, Functional Characterization, Chromosomal Localization, and Gene Structure of Junctate, a Novel Integral Calcium Binding Protein of Sarco(Endo)Plasmic Reticulum Membrane. J. Biol. Chem..

[65] Divet A., Paesante S., Grasso C., Cavagna D., Tiveron C., Paolini C., Protasi F., Huchet-Cadiou C., Treves S., Zorzato F. (2007). Increased Ca^2+^ Storage Capacity of the Skeletal Muscle Sarcoplasmic Reticulum of Transgenic Mice Over-Expressing Membrane Bound Calcium Binding Protein Junctate. J. Cell. Physiol..

[66] Srikanth S., Jew M., Kim K.-D., Yee M.-K., Abramson J., Gwack Y. (2012). Junctate Is a Ca^2+^-Sensing Structural Component of Orai1 and Stromal Interaction Molecule 1 (STIM1). Proc. Natl. Acad. Sci. USA.

[67] Shin D.W., Pan Z., Kim E.K., Lee J.M., Bhat M.B., Parness J., Kim D.H., Ma J. (2003). A Retrograde Signal from Calsequestrin for the Regulation of Store-Operated Ca^2+^ Entry in Skeletal Muscle. J. Biol. Chem..

[68] Zhao X., Min C.K., Ko J.-K., Parness J., Kim D.H., Weisleder N., Ma J. (2010). Increased Store-Operated Ca^2+^ Entry in Skeletal Muscle with Reduced Calsequestrin-1 Expression. Biophys. J..

[69] Wang L., Zhang L., Li S., Zheng Y., Yan X., Chen M., Wang H., Putney J.W., Luo D. (2015). Retrograde Regulation of STIM1-Orai1 Interaction and Store-Operated Ca^2+^ Entry by Calsequestrin. Sci. Rep..

[70] Zhang L., Wang L., Li S., Xue J., Luo D. (2016). Calsequestrin-1 Regulates Store-Operated Ca^2+^ Entry by Inhibiting STIM1 Aggregation. Cell. Physiol. Biochem..

[71] Shimuta M., Komazaki S., Nishi M., Iino M., Nakagawara K., Takeshima H. (1998). Structure and Expression of Mitsugumin29 Gene. Febs Lett..

[72] Nishi M., Komazaki S., Kurebayashi N., Ogawa Y., Noda T., Iino M., Takeshima H. (1999). Abnormal Features in Skeletal Muscle from Mice Lacking Mitsugumin29. J. Cell. Biol..

[73] Zhao X., Weisleder N., Thornton A., Oppong Y., Campbell R., Ma J., Brotto M. (2008). Compromised Store-Operated Ca ^2^ Entry in Aged Skeletal Muscle. Aging Cell.

[74] Nagaraj R.Y., Nosek C.M., Brotto M.A., Nishi M., Takeshima H., Nosek T.M., Ma J. (2000). Increased Susceptibility to Fatigue of Slow- and Fast-Twitch Muscles from Mice Lacking the MG29 Gene. Physiol Genom..

[75] Kurebayashi N., Takeshima H., Nishi M., Murayama T., Suzuki E., Ogawa Y. (2003). Changes in Ca^2+^ Handling in Adult MG29-Deficient Skeletal Muscle. Biochem. Biophys. Res. Commun..

[76] Edwards J.N., Blackmore D.G., Gilbert D.F., Murphy R.M., Launikonis B.S. (2011). Store-Operated Calcium Entry Remains Fully Functional in Aged Mouse Skeletal Muscle despite a Decline in STIM1 Protein Expression: Store-Operated Ca^2+^ Entry in Aged Skeletal Muscle. Aging Cell.

[77] Kang F., Zhou M., Huang X., Fan J., Wei L., Boulanger J., Liu Z., Salamero J., Liu Y., Chen L. (2019). E-Syt1 Re-Arranges STIM1 Clusters to Stabilize Ring-Shaped ER-PM Contact Sites and Accelerate Ca^2+^ Store Replenishment. Sci. Rep..

[78] Berlansky S., Humer C., Sallinger M., Frischauf I. (2021). More Than Just Simple Interaction between STIM and Orai Proteins: CRAC Channel Function Enabled by a Network of Interactions with Regulatory Proteins. IJMS.

[79] Derler I., Jardin I., Stathopulos P.B., Muik M., Fahrner M., Zayats V., Pandey S.K., Poteser M., Lackner B., Absolonova M. (2016). Cholesterol Modulates Orai1 Channel Function. Sci. Signal..

[80] Rosemblatt M., Hidalgo C., Vergara C., Ikemoto N. (1981). Immunological and Biochemical Properties of Transverse Tubule Membranes Isolated from Rabbit Skeletal Muscle. J. Biol. Chem..

[81] Carozzi A.J., Ikonen E., Lindsay M.R., Parton R.G. (2000). Role of Cholesterol in Developing T-Tubules: Analogous Mechanisms for T-Tubule and Caveolae Biogenesis. Traffic.

[82] Maléth J., Choi S., Muallem S., Ahuja M. (2014). Translocation between PI(4,5)P2-Poor and PI(4,5)P2-Rich Microdomains during Store Depletion Determines STIM1 Conformation and Orai1 Gating. Nat. Commun..

[83] Allard B., Couchoux H., Pouvreau S., Jacquemond V. (2006). Sarcoplasmic Reticulum Ca ^2+^ Release and Depletion Fail to Affect Sarcolemmal Ion Channel Activity in Mouse Skeletal Muscle: Ca^2+^ Release and Ion Channel Activity in Skeletal Muscle. J. Physiol..

[84] Protasi F., Pietrangelo L., Boncompagni S. (2020). Calcium Entry Units (CEUs): Perspectives in Skeletal Muscle Function and Disease. J. Muscle Res. Cell. Motil..

[85] Michelucci A., Boncompagni S., Pietrangelo L., García-Castañeda M., Takano T., Malik S., Dirksen R.T., Protasi F. (2019). Transverse Tubule Remodeling Enhances Orai1-Dependent Ca^2+^ Entry in Skeletal Muscle. eLife.

[86] Silva-Rojas R., Laporte J., Böhm J. (2020). STIM1/ORAI1 Loss-of-Function and Gain-of-Function Mutations Inversely Impact on SOCE and Calcium Homeostasis and Cause Multi-Systemic Mirror Diseases. Front. Physiol..

[87] Michelucci A., Boncompagni S., Pietrangelo L., Takano T., Protasi F., Dirksen R.T. (2020). Pre-Assembled Ca2+ Entry Units and Constitutively Active Ca^2+^ Entry in Skeletal Muscle of Calsequestrin-1 Knockout Mice. J. Physiol..

[88] Jayasinghe I.D., Munro M., Baddeley D., Launikonis B.S., Soeller C. (2014). Observation of the Molecular Organization of Calcium Release Sites in Fast- and Slow-Twitch Skeletal Muscle with Nanoscale Imaging. J. R Soc. Interface..

[89] Lyfenko A.D., Dirksen R.T. (2008). Differential Dependence of Store-Operated and Excitation-Coupled Ca^2+^ Entry in Skeletal Muscle on STIM1 and Orai1: Differential Dependence of SOCE and ECCE on STIM1 and Orai1. J. Physiol..

[90] Cully T.R., Choi R.H., Bjorksten A.R., Stephenson D.G., Murphy R.M., Launikonis B.S. (2018). Junctional Membrane Ca^2+^ Dynamics in Human Muscle Fibers Are Altered by Malignant Hyperthermia Causative RyR Mutation. Proc. Natl. Acad. Sci. USA.

[91] Boncompagni S., Thomas M., Lopez J.R., Allen P.D., Yuan Q., Kranias E.G., Franzini-Armstrong C., Perez C.F. (2012). Triadin/Junctin Double Null Mouse Reveals a Differential Role for Triadin and Junctin in Anchoring CASQ to the JSR and Regulating Ca(^2+^) Homeostasis. PLoS ONE.

[92] Sébastien M., Giannesini B., Aubin P., Brocard J., Chivet M., Pietrangelo L., Boncompagni S., Bosc C., Brocard J., Rendu J. (2018). Deletion of the Microtubule-Associated Protein 6 (MAP6) Results in Skeletal Muscle Dysfunction. Skelet. Muscle.

[93] Cully T.R., Murphy R.M., Roberts L., Raastad T., Fassett R.G., Coombes J.S., Jayasinghe I.D., Launikonis B.S. (2017). Human Skeletal Muscle Plasmalemma Alters Its Structure to Change Its Ca^2+^-Handling Following Heavy-Load Resistance Exercise. Nat. Commun..

[94] Launikonis B.S., Barnes M., Stephenson D.G. (2003). Identification of the Coupling between Skeletal Muscle Store-Operated Ca^2+^ Entry and the Inositol Trisphosphate Receptor. Proc. Natl. Acad. Sci. USA.

[95] Launikonis B.S., Ríos E. (2007). Store-Operated Ca^2+^ Entry during Intracellular Ca^2+^ Release in Mammalian Skeletal Muscle: SOCE during Ca ^2+^ Release in Muscle. J. Physiol..

[96] Launikonis B.S., Stephenson D.G., Friedrich O. (2009). Rapid Ca^2+^ Flux through the Transverse Tubular Membrane, Activated by Individual Action Potentials in Mammalian Skeletal Muscle: Action Potential-Activated Ca^2+^ Flux. J. Physiol..

[97] Cully T.R., Edwards J.N., Murphy R.M., Launikonis B.S. (2016). A Quantitative Description of Tubular System Ca^2+^ Handling in Fast- and Slow-Twitch Muscle Fibres: Quantitating t-System Ca^2+^ Handling. J. Physiol..

[98] Stienen G.J.M. (2000). Chronicle of Skinned Muscle Fibres. J. Physiol..

[99] Posterino G.S. (2001). “Current” Advances in Mechanically Skinned Skeletal Muscle Fibres. Clin. Exp. Pharmacol. Physiol..

[100] Lamb G.D., Stephenson D.G. (2018). Measurement of Force and Calcium Release Using Mechanically Skinned Fibers from Mammalian Skeletal Muscle. J. Appl. Physiol..

[101] Endo M. (1964). ENTRY OF A DYE INTO THE SARCOTUBULAR SYSTEM OF MUSCLE. Nature.

[102] Lamb G.D., Junankar P.R., Stephenson D.G. (1995). Raised Intracellular [Ca^2+^] Abolishes Excitation-Contraction Coupling in Skeletal Muscle Fibres of Rat and Toad. J. Physiol..

[103] Lamb G.D., Stephenson D.G. (1990). Calcium Release in Skinned Muscle Fibres of the Toad by Transverse Tubule Depolarization or by Direct Stimulation. J. Physiol..

[104] Posterino G.S., Lamb G.D. (2003). Effect of Sarcoplasmic Reticulum Ca^2+^ Content on Action Potential-Induced Ca^2+^ Release in Rat Skeletal Muscle Fibres. J. Physiol..

[105] Posterino G.S., Lamb G.D., Stephenson D.G. (2000). Twitch and Tetanic Force Responses and Longitudinal Propagation of Action Potentials in Skinned Skeletal Muscle Fibres of the Rat. J. Physiol..

[106] Lamb G.D., Cellini M.A., Stephenson D.G. (2001). Different Ca^2+^ Releasing Action of Caffeine and Depolarisation in Skeletal Muscle Fibres of the Rat. J. Physiol..

[107] Lamb G.D., Stephenson D.G. (1994). Effects of Intracellular PH and [Mg^2+^] on Excitation-Contraction Coupling in Skeletal Muscle Fibres of the Rat. J. Physiol..

[108] Cully T.R., Edwards J.N., Launikonis B.S. (2014). Activation and Propagation of Ca^2+^ Release from inside the Sarcoplasmic Reticulum Network of Mammalian Skeletal Muscle: Ca^2+^ Waves in Mammalian Muscle. J. Physiol..

[109] Edwards J.N., Murphy R.M., Cully T.R., von Wegner F., Friedrich O., Launikonis B.S. (2010). Ultra-Rapid Activation and Deactivation of Store-Operated Ca^2+^ Entry in Skeletal Muscle. Cell Calcium.

[110] Li H., Ding X., Lopez J.R., Takeshima H., Ma J., Allen P.D., Eltit J.M. (2010). Impaired Orai1-Mediated Resting Ca^2+^ Entry Reduces the Cytosolic [Ca ^2+^ ] and Sarcoplasmic Reticulum Ca^2+^ Loading in Quiescent Junctophilin 1 Knock-out Myotubes. J. Biol. Chem..

[111] Koenig X., Choi R.H., Schicker K., Singh D.P., Hilber K., Launikonis B.S. (2019). Mechanistic Insights into Store-Operated Ca^2+^ Entry during Excitation-Contraction Coupling in Skeletal Muscle. Biochim. Biophys. Acta (BBA)—Mol. Cell Res..

[112] Rudolf R., Magalhães P.J., Pozzan T. (2006). Direct in Vivo Monitoring of Sarcoplasmic Reticulum Ca^2+^ and Cytosolic CAMP Dynamics in Mouse Skeletal Muscle. J. Cell. Biol..

[113] Lamb G.D., Stephenson D.G. (1991). Effect of Mg^2+^ on the Control of Ca^2+^ Release in Skeletal Muscle Fibres of the Toad. J. Physiol..

[114] Choi R.H., Koenig X., Launikonis B.S. (2017). Dantrolene Requires Mg^2+^ to Arrest Malignant Hyperthermia. Proc. Natl. Acad. Sci. USA.

[115] Manno C., Sztretye M., Figueroa L., Allen P.D., Ríos E. (2013). Dynamic Measurement of the Calcium Buffering Properties of the Sarcoplasmic Reticulum in Mouse Skeletal Muscle: Ca^2+^ Buffering in the SR. J. Physiol..

[116] Luik R.M., Wang B., Prakriya M., Wu M.M., Lewis R.S. (2008). Oligomerization of STIM1 Couples ER Calcium Depletion to CRAC Channel Activation. Nature.

[117] Soboloff J., Rothberg B.S., Madesh M., Gill D.L. (2012). STIM Proteins: Dynamic Calcium Signal Transducers. Nat. Rev. Mol. Cell. Biol..

[118] MacLennan D.H., Wong P.T.S. (1971). Isolation of a Calcium-Sequestering Protein from Sarcoplasmic Reticulum. Proc. Natl. Acad. Sci. USA.

[119] Ikemoto N., Bhatnagar G.M., Nagy B., Gergely J. (1972). Interaction of Divalent Cations with the 55,000-Dalton Protein Component of the Sarcoplasmic Reticulum: Studies of fluorescence and circular dichroism. J. Biol. Chem..

[120] Murphy R.M., Larkins N.T., Mollica J.P., Beard N.A., Lamb G.D. (2009). Calsequestrin Content and SERCA Determine Normal and Maximal Ca^2+^ Storage Levels in Sarcoplasmic Reticulum of Fast- and Slow-Twitch Fibres of Rat. J. Physiol..

[121] Beard N.A., Laver D.R., Dulhunty A.F. (2004). Calsequestrin and the Calcium Release Channel of Skeletal and Cardiac Muscle. Prog. Biophys. Mol. Biol.

[122] Royer L., Ríos E. (2009). Deconstructing Calsequestrin. Complex Buffering in the Calcium Store of Skeletal Muscle. J. Physiol..

[123] Woo J.S., Jeong S.Y., Park J.H., Choi J.H., Lee E.H. (2020). Calsequestrin: A Well-Known but Curious Protein in Skeletal Muscle. Exp. Mol. Med..

[124] MacLennan D.H., Reithmeier R.A. (1998). Ion Tamers. Nat. Struct. Biol..

[125] Manno C., Figueroa L.C., Gillespie D., Fitts R., Kang C., Franzini-Armstrong C., Rios E. (2017). Calsequestrin Depolymerizes When Calcium Is Depleted in the Sarcoplasmic Reticulum of Working Muscle. Proc. Natl. Acad. Sci. USA.

[126] Ivarsson N., Mattsson C.M., Cheng A.J., Bruton J.D., Ekblom B., Lanner J.T., Westerblad H. (2019). SR Ca^2+^ Leak in Skeletal Muscle Fibers Acts as an Intracellular Signal to Increase Fatigue Resistance. J. Gen. Physiol..

[127] Bellinger A.M., Reiken S., Carlson C., Mongillo M., Liu X., Rothman L., Matecki S., Lacampagne A., Marks A.R. (2009). Hypernitrosylated Ryanodine Receptor Calcium Release Channels Are Leaky in Dystrophic Muscle. Nat. Med..

[128] Reddish F.N., Miller C.L., Deng X., Dong B., Patel A.A., Ghane M.A., Mosca B., McBean C., Wu S., Solntsev K.M. (2021). Rapid Subcellular Calcium Responses and Dynamics by Calcium Sensor G-CatchER+. iScience.

[129] Carrell E.M., Coppola A.R., McBride H.J., Dirksen R.T. (2016). Orai1 Enhances Muscle Endurance by Promoting Fatigue-Resistant Type I Fiber Content but Not through Acute Store-Operated Ca^2+^ Entry. FASEB J..

[130] Lee E.H., Cherednichenko G., Pessah I.N., Allen P.D. (2006). Functional Coupling between TRPC3 and RyR1 Regulates the Expressions of Key Triadic Proteins. J. Biol. Chem..

[131] Dirksen R.T. (2009). Checking Your SOCCs and Feet: The Molecular Mechanisms of Ca^2+^ Entry in Skeletal Muscle: Ca^2+^ Entry in Skeletal Muscle. J. Physiol..

[132] Liao Y., Erxleben C., Abramowitz J., Flockerzi V., Zhu M.X., Armstrong D.L., Birnbaumer L. (2008). Functional Interactions among Orai1, TRPCs, and STIM1 Suggest a STIM-Regulated Heteromeric Orai/TRPC Model for SOCE/Icrac Channels. Proc. Natl. Acad. Sci. USA.

[133] Antigny F., Sabourin J., Saüc S., Bernheim L., Koenig S., Frieden M. (2017). TRPC1 and TRPC4 Channels Functionally Interact with STIM1L to Promote Myogenesis and Maintain Fast Repetitive Ca^2+^ Release in Human Myotubes. Biochim. Biophys. Acta (BBA)—Mol. Cell Res..

[134] Cheng K.T., Liu X., Ong H.L., Ambudkar I.S. (2008). Functional Requirement for Orai1 in Store-Operated TRPC1-STIM1 Channels. J. Biol. Chem..

[135] Ong H.L., Cheng K.T., Liu X., Bandyopadhyay B.C., Paria B.C., Soboloff J., Pani B., Gwack Y., Srikanth S., Singh B.B. (2007). Dynamic Assembly of TRPC1-STIM1-Orai1 Ternary Complex Is Involved in Store-Operated Calcium Influx. Evidence for Similarities in Store-Operated and Calcium Release-Activated Calcium Channel Components. J. Biol. Chem..

[136] Jardin I., Lopez J.J., Salido G.M., Rosado J.A. (2008). Orai1 Mediates the Interaction between STIM1 and HTRPC1 and Regulates the Mode of Activation of HTRPC1-Forming Ca^2+^ Channels. J. Biol. Chem..

[137] Berridge M.J. (1995). Capacitative Calcium Entry. Biochem. J..

[138] Sampieri A., Diaz-Muñoz M., Antaramian A., Vaca L. (2005). The Foot Structure from the Type 1 Ryanodine Receptor Is Required for Functional Coupling to Store-Operated Channels. J. Biol. Chem..

[139] Kiselyov K.I., Shin D.M., Wang Y., Pessah I.N., Allen P.D., Muallem S. (2000). Gating of Store-Operated Channels by Conformational Coupling to Ryanodine Receptors. Mol. Cell..

[140] Estrada M., Espinosa A., Gibson C.J., Uhlen P., Jaimovich E. (2005). Capacitative Calcium Entry in Testosterone-Induced Intracellular Calcium Oscillations in Myotubes. J. Endocrinol..

[141] Powell J.A., Carrasco M.A., Adams D.S., Drouet B., Rios J., Müller M., Estrada M., Jaimovich E. (2001). IP(3) Receptor Function and Localization in Myotubes: An Unexplored Ca^2+^ Signaling Pathway in Skeletal Muscle. J. Cell. Sci..

[142] Lilliu E., Hilber K., Launikonis B.S., Koenig X. (2020). Phasic Store-Operated Ca^2+^ Entry During Excitation-Contraction Coupling in Skeletal Muscle Fibers From Exercised Mice. Front. Physiol..

[143] Baylor S.M., Hollingworth S. (2003). Sarcoplasmic Reticulum Calcium Release Compared in Slow-Twitch and Fast-Twitch Fibres of Mouse Muscle. J. Physiol..

[144] Adams R.J., Schwartz A. (1980). Comparative Mechanisms for Contraction of Cardiac and Skeletal Muscle. Chest.

[145] Vergara J.L., DiFranco M., Novo D., Bearman G.H., Bornhop D.J., Levenson R.M. (2001). Dimensions of Calcium Release Domains in Frog Skeletal Muscle Fibers.

[146] Darbellay B., Arnaudeau S., Bader C.R., Konig S., Bernheim L. (2011). STIM1L Is a New Actin-Binding Splice Variant Involved in Fast Repetitive Ca^2+^ Release. J. Cell. Biol..

[147] Merritt J.E., Armstrong W.P., Benham C.D., Hallam T.J., Jacob R., Jaxa-Chamiec A., Leigh B.K., McCarthy S.A., Moores K.E., Rink T.J. (1990). SK&F 96365, a Novel Inhibitor of Receptor-Mediated Calcium Entry. Biochem. J..

[148] Singh A., Hildebrand M.E., Garcia E., Snutch T.P. (2010). The Transient Receptor Potential Channel Antagonist SKF96365 Is a Potent Blocker of Low-Voltage-Activated T-Type Calcium Channels. Br. J. Pharmacol..

[149] Maruyama T., Kanaji T., Nakade S., Kanno T., Mikoshiba K. (1997). 2APB, 2-Aminoethoxydiphenyl Borate, a Membrane-Penetrable Modulator of Ins(1,4,5)P3-Induced Ca^2+^ Release. J. Biochem..

[150] Djuric S.W., BaMaung N.Y., Basha A., Liu H., Luly J.R., Madar D.J., Sciotti R.J., Tu N.P., Wagenaar F.L., Wiedeman P.E. (2000). 3,5-Bis(Trifluoromethyl)Pyrazoles: A Novel Class of NFAT Transcription Factor Regulator. J. Med. Chem..

[151] Trevillyan J.M., Chiou X.G., Chen Y.W., Ballaron S.J., Sheets M.P., Smith M.L., Wiedeman P.E., Warrior U., Wilkins J., Gubbins E.J. (2001). Potent Inhibition of NFAT Activation and T Cell Cytokine Production by Novel Low Molecular Weight Pyrazole Compounds. J. Biol. Chem..

[152] Chen Y.-W., Smith M.L., Chiou G.X., Ballaron S., Sheets M.P., Gubbins E., Warrior U., Wilkins J., Surowy C., Nakane M. (2002). TH1 and TH2 Cytokine Inhibition by 3,5-Bis(Trifluoromethyl)Pyrazoles, a Novel Class of Immunomodulators. Cell. Immunol..

[153] Putney J.W. (2010). Pharmacology of Store-Operated Calcium Channels. Mol. Interv..

[154] Bakowski D., Murray F., Parekh A.B. (2021). Store-Operated Ca^2+^ Channels: Mechanism, Function, Pharmacology, and Therapeutic Targets. Annu. Rev. Pharmacol. Toxicol..

[155] Tian C., Du L., Zhou Y., Li M. (2016). Store-Operated CRAC Channel Inhibitors: Opportunities and Challenges. Future Med. Chem..

[156] Zhang X., Xin P., Yoast R.E., Emrich S.M., Johnson M.T., Pathak T., Benson J.C., Azimi I., Gill D.L., Monteith G.R. (2020). Distinct Pharmacological Profiles of ORAI1, ORAI2, and ORAI3 Channels. Cell Calcium.

[157] Vassilopoulos S., Brocard J., Garcia L., Marty I., Bouron A. (2007). Retrograde Regulation of Store-Operated Calcium Channels by the Ryanodine Receptor-Associated Protein Triadin 95 in Rat Skeletal Myotubes. Cell Calcium.

[158] Eltit J.M., Ding X., Pessah I.N., Allen P.D., Lopez J.R. (2013). Nonspecific Sarcolemmal Cation Channels Are Critical for the Pathogenesis of Malignant Hyperthermia. FASEB J..

[159] Gutierrez-Martin Y., Martin-Romero F.J., Henao F. (2005). Store-Operated Calcium Entry in Differentiated C2C12 Skeletal Muscle Cells. Biochim. Biophys. Acta.

[160] Vazquez G., de Boland A.R., Boland R.L. (1998). 1alpha,25-Dihydroxy-Vitamin-D3-Induced Store-Operated Ca^2+^ Influx in Skeletal Muscle Cells. Modulation by Phospholipase c, Protein Kinase c, and Tyrosine Kinases. J. Biol. Chem..

[161] Reichling D.B., MacDermott A.B. (1991). Lanthanum Actions on Excitatory Amino Acid-Gated Currents and Voltage-Gated Calcium Currents in Rat Dorsal Horn Neurons. J. Physiol..

[162] Leffler A., Linte R.M., Nau C., Reeh P., Babes A. (2007). A High-Threshold Heat-Activated Channel in Cultured Rat Dorsal Root Ganglion Neurons Resembles TRPV2 and Is Blocked by Gadolinium. Eur. J. Neurosci..

[163] Oz M., Tchugunova Y.B., Dunn S.M. (2001). Direct Inhibition of Voltage-Dependent Ca^2+^ Fluxes by Ethanol and Higher Alcohols in Rabbit T-Tubule Membranes. Eur. J. Pharmacol..

[164] Lee E.H., Lopez J.R., Li J., Protasi F., Pessah I.N., Kim D.H., Allen P.D. (2004). Conformational Coupling of DHPR and RyR1 in Skeletal Myotubes Is Influenced by Long-Range Allosterism: Evidence for a Negative Regulatory Module. Am. J. Physiol. Cell. Physiol..

[165] Mosca B., Eckhardt J., Bergamelli L., Treves S., Bongianino R., De Negri M., Priori S.G., Protasi F., Zorzato F. (2016). Role of the JP45-Calsequestrin Complex on Calcium Entry in Slow Twitch Skeletal Muscles. J. Biol. Chem..

[166] Bannister R.A., Pessah I.N., Beam K.G. (2009). The Skeletal L-Type Ca ^2+^ Current Is a Major Contributor to Excitation-Coupled Ca ^2+^ Entry. J. Gen. Physiol..

[167] Chung S.C., McDonald T.V., Gardner P. (1994). Inhibition by SK&F 96365 of Ca^2+^ Current, IL-2 Production and Activation in T Lymphocytes. Br. J. Pharmacol..

[168] Olivera J.F., Fernando Olivera J., Pizarro G. (2010). Two Inhibitors of Store Operated Ca^2+^ Entry Suppress Excitation Contraction Coupling in Frog Skeletal Muscle. J. Muscle Res. Cell. Motil..

[169] Ho T.C., Horn N.A., Huynh T., Kelava L., Lansman J.B. (2012). Evidence TRPV4 Contributes to Mechanosensitive Ion Channels in Mouse Skeletal Muscle Fibers. Channels.

[170] Tanahashi Y., Wang B., Murakami Y., Unno T., Matsuyama H., Nagano H., Komori S. (2016). Inhibitory Effects of SKF96365 on the Activities of K(+) Channels in Mouse Small Intestinal Smooth Muscle Cells. J. Vet. Med. Sci..

[171] Iouzalen L., Lantoine F., Pernollet M.G., Millanvoye-Van Brussel E., Devynck M.A., David-Dufilho M. (1996). SK&F 96365 Inhibits Intracellular Ca^2+^ Pumps and Raises Cytosolic Ca^2+^ Concentration without Production of Nitric Oxide and von Willebrand Factor. Cell Calcium.

[172] Song M., Chen D., Yu S.P. (2014). The TRPC Channel Blocker SKF 96365 Inhibits Glioblastoma Cell Growth by Enhancing Reverse Mode of the Na(+) /Ca(^2+^) Exchanger and Increasing Intracellular Ca(^2+^). Br. J. Pharmacol..

[173] Cherednichenko G., Hurne A.M., Fessenden J.D., Lee E.H., Allen P.D., Beam K.G., Pessah I.N. (2004). Conformational Activation of Ca^2+^ Entry by Depolarization of Skeletal Myotubes. Proc. Natl. Acad. Sci. USA.

[174] Lanner J.T., Katz A., Tavi P., Sandström M.E., Zhang S.-J., Wretman C., James S., Fauconnier J., Lännergren J., Bruton J.D. (2006). The Role of Ca^2+^ Influx for Insulin-Mediated Glucose Uptake in Skeletal Muscle. Diabetes.

[175] Juretić N., Jorquera G., Caviedes P., Jaimovich E., Riveros N. (2012). Electrical Stimulation Induces Calcium-Dependent up-Regulation of Neuregulin-1β in Dystrophic Skeletal Muscle Cell Lines. Cell Physiol. Biochem..

[176] Missiaen L., Callewaert G., De Smedt H., Parys J.B. (2001). 2-Aminoethoxydiphenyl Borate Affects the Inositol 1,4,5-Trisphosphate Receptor, the Intracellular Ca^2+^ Pump and the Non-Specific Ca^2+^ Leak from the Non-Mitochondrial Ca^2+^ Stores in Permeabilized A7r5 Cells. Cell Calcium.

[177] Carrasco M.A., Riveros N., Ríos J., Müller M., Torres F., Pineda J., Lantadilla S., Jaimovich E. (2003). Depolarization-Induced Slow Calcium Transients Activate Early Genes in Skeletal Muscle Cells. Am. J. Physiol. Cell. Physiol..

[178] Liberona J.L., Cárdenas J.C., Reyes R., Hidalgo J., Molgó J., Jaimovich E. (2008). Sodium-Dependent Action Potentials Induced by Brevetoxin-3 Trigger Both IP3 Increase and Intracellular Ca^2+^ Release in Rat Skeletal Myotubes. Cell. Calcium..

[179] Gregory R.B., Rychkov G., Barritt G.J. (2001). Evidence That 2-Aminoethyl Diphenylborate Is a Novel Inhibitor of Store-Operated Ca^2+^ Channels in Liver Cells, and Acts through a Mechanism Which Does Not Involve Inositol Trisphosphate Receptors. Biochem. J..

[180] Prakriya M., Lewis R.S. (2001). Potentiation and Inhibition of Ca(^2+^) Release-Activated Ca(^2+^) Channels by 2-Aminoethyldiphenyl Borate (2-APB) Occurs Independently of IP(3) Receptors. J. Physiol..

[181] Bootman M.D., Collins T.J., Mackenzie L., Roderick H.L., Berridge M.J., Peppiatt C.M. (2002). 2-Aminoethoxydiphenyl Borate (2-APB) Is a Reliable Blocker of Store-Operated Ca^2+^ Entry but an Inconsistent Inhibitor of InsP3-Induced Ca^2+^ Release. Faseb J..

[182] DeHaven W.I., Smyth J.T., Boyles R.R., Bird G.S., Putney J.W. (2008). Complex Actions of 2-Aminoethyldiphenyl Borate on Store-Operated Calcium Entry. J. Biol. Chem..

[183] Peinelt C., Lis A., Beck A., Fleig A., Penner R. (2008). 2-Aminoethoxydiphenyl Borate Directly Facilitates and Indirectly Inhibits STIM1-Dependent Gating of CRAC Channels. J. Physiol..

[184] Lis A., Peinelt C., Beck A., Parvez S., Monteilh-Zoller M., Fleig A., Penner R. (2007). CRACM1, CRACM2, and CRACM3 Are Store-Operated Ca^2+^ Channels with Distinct Functional Properties. Curr. Biol..

[185] Ishikawa J., Ohga K., Yoshino T., Takezawa R., Ichikawa A., Kubota H., Yamada T. (2003). A Pyrazole Derivative, YM-58483, Potently Inhibits Store-Operated Sustained Ca^2+^ Influx and IL-2 Production in T Lymphocytes. J. Immunol..

[186] Zitt C., Strauss B., Schwarz E.C., Spaeth N., Rast G., Hatzelmann A., Hoth M. (2004). Potent Inhibition of Ca^2+^ Release-Activated Ca^2+^ Channels and T-Lymphocyte Activation by the Pyrazole Derivative BTP2. J. Biol. Chem..

[187] Steinckwich N., Frippiat J.-P., Stasia M.-J., Erard M., Boxio R., Tankosic C., Doignon I., Nüsse O. (2007). Potent Inhibition of Store-Operated Ca^2+^ Influx and Superoxide Production in HL60 Cells and Polymorphonuclear Neutrophils by the Pyrazole Derivative BTP2. J. Leukoc. Biol..

[188] Takezawa R., Cheng H., Beck A., Ishikawa J., Launay P., Kubota H., Kinet J.-P., Fleig A., Yamada T., Penner R. (2006). A Pyrazole Derivative Potently Inhibits Lymphocyte Ca^2+^ Influx and Cytokine Production by Facilitating Transient Receptor Potential Melastatin 4 Channel Activity. Mol. Pharmacol..

[189] He L.-P., Hewavitharana T., Soboloff J., Spassova M.A., Gill D.L. (2005). A Functional Link between Store-Operated and TRPC Channels Revealed by the 3,5-Bis(Trifluoromethyl)Pyrazole Derivative, BTP2. J. Biol. Chem..

[190] Thornton A.M., Zhao X., Weisleder N., Brotto L.S., Bougoin S., Nosek T.M., Reid M., Hardin B., Pan Z., Ma J. (2011). Store-Operated Ca^2+^ Entry (SOCE) Contributes to Normal Skeletal Muscle Contractility in Young but Not in Aged Skeletal Muscle. Aging.

[191] Zhao X., Moloughney J.G., Zhang S., Komazaki S., Weisleder N. (2012). Orai1 Mediates Exacerbated Ca^2+^ Entry in Dystrophic Skeletal Muscle. PLoS ONE.

[192] Meizoso-Huesca A., Launikonis B.S. (2021). The Orai1 Inhibitor BTP2 Has Multiple Effects on Ca^2+^ Handling in Skeletal Muscle. J. Gen. Physiol..

[193] Ashmole I., Duffy S.M., Leyland M.L., Morrison V.S., Begg M., Bradding P. (2012). CRACM/Orai Ion Channel Expression and Function in Human Lung Mast Cells. J. Allergy Clin. Immunol..

[194] Derler I., Schindl R., Fritsch R., Heftberger P., Riedl M.C., Begg M., House D., Romanin C. (2013). The Action of Selective CRAC Channel Blockers Is Affected by the Orai Pore Geometry. Cell Calcium.

[195] Rice L.V., Bax H.J., Russell L.J., Barrett V.J., Walton S.E., Deakin A.M., Thomson S.A., Lucas F., Solari R., House D. (2013). Characterization of Selective Calcium-Release Activated Calcium Channel Blockers in Mast Cells and T-Cells from Human, Rat, Mouse and Guinea-Pig Preparations. Eur. J. Pharmacol..

[196] Bulla M., Gyimesi G., Kim J.H., Bhardwaj R., Hediger M.A., Frieden M., Demaurex N. (2019). ORAI1 Channel Gating and Selectivity Is Differentially Altered by Natural Mutations in the First or Third Transmembrane Domain. J. Physiol..

[197] Waldherr L., Tiffner A., Mishra D., Sallinger M., Schober R., Frischauf I., Schmidt T., Handl V., Sagmeister P., Köckinger M. (2020). Blockage of Store-Operated Ca^2+^ Influx by Synta66 Is Mediated by Direct Inhibition of the Ca^2+^ Selective Orai1 Pore. Cancers.

[198] Li J., McKeown L., Ojelabi O., Stacey M., Foster R., O’Regan D., Porter K.E., Beech D.J. (2011). Nanomolar Potency and Selectivity of a Ca^2+^ Release-Activated Ca^2+^ Channel Inhibitor against Store-Operated Ca^2+^ Entry and Migration of Vascular Smooth Muscle Cells. Br. J. Pharmacol..

[199] Di Sabatino A., Rovedatti L., Kaur R., Spencer J.P., Brown J.T., Morisset V.D., Biancheri P., Leakey N.A.B., Wilde J.I., Scott L. (2009). Targeting Gut T Cell Ca^2+^ Release-Activated Ca^2+^ Channels Inhibits T Cell Cytokine Production and T-Box Transcription Factor T-Bet in Inflammatory Bowel Disease. J. Immunol..

[200] Riva B., Griglio A., Serafini M., Cordero-Sanchez C., Aprile S., Di Paola R., Gugliandolo E., Alansary D., Biocotino I., Lim D. (2018). Pyrtriazoles, a Novel Class of Store-Operated Calcium Entry Modulators: Discovery, Biological Profiling, and in Vivo Proof-of-Concept Efficacy in Acute Pancreatitis. J. Med. Chem..

[201] Serafini M., Cordero-Sanchez C., Di Paola R., Bhela I.P., Aprile S., Purghè B., Fusco R., Cuzzocrea S., Genazzani A.A., Riva B. (2020). Store-Operated Calcium Entry as a Therapeutic Target in Acute Pancreatitis: Discovery and Development of Drug-Like SOCE Inhibitors. J. Med. Chem..

[202] Azimi I., Stevenson R.J., Zhang X., Meizoso-Huesca A., Xin P., Johnson M., Flanagan J.U., Chalmers S.B., Yoast R.E., Kapure J.S. (2020). A New Selective Pharmacological Enhancer of the Orai1 Ca^2+^ Channel Reveals Roles for Orai1 in Smooth and Skeletal Muscle Functions. ACS Pharmacol. Transl. Sci..

[203] Thomas N.L., Williams A.J. (2012). Pharmacology of Ryanodine Receptors and Ca^2+^-Induced Ca^2+^ Release. Wires Membr. Transp. Signal..

[204] Horinouchi T., Higashi T., Higa T., Terada K., Mai Y., Aoyagi H., Hatate C., Nepal P., Horiguchi M., Harada T. (2012). Different Binding Property of STIM1 and Its Novel Splice Variant STIM1L to Orai1, TRPC3, and TRPC6 Channels. Biochem. Biophys. Res. Commun..

[205] Saüc S., Bulla M., Nunes P., Orci L., Marchetti A., Antigny F., Bernheim L., Cosson P., Frieden M., Demaurex N. (2015). STIM1L Traps and Gates Orai1 Channels without Remodeling the Cortical ER. J. Cell. Sci..

[206] Dyrda A., Koenig S., Frieden M. (2020). STIM1 Long and STIM1 Gate Differently TRPC1 during Store-Operated Calcium Entry. Cell Calcium.

[207] Antigny F., Koenig S., Bernheim L., Frieden M. (2013). During Post-Natal Human Myogenesis, Normal Myotube Size Requires TRPC1- and TRPC4-Mediated Ca^2+^ Entry. J. Cell. Sci..

[208] Saüc S., Frieden M. (2017). Neurological and Motor Disorders: TRPC in the Skeletal Muscle. Adv. Exp. Med. Biol..

[209] Darbellay B., Arnaudeau S., Ceroni D., Bader C.R., Konig S., Bernheim L. (2010). Human Muscle Economy Myoblast Differentiation and Excitation-Contraction Coupling Use the Same Molecular Partners, STIM1 and STIM2. J. Biol. Chem..

[210] Brandman O., Liou J., Park W.S., Meyer T. (2007). STIM2 Is a Feedback Regulator That Stabilizes Basal Cytosolic and Endoplasmic Reticulum Ca^2+^ Levels. Cell.

[211] Berna-Erro A., Braun A., Kraft R., Kleinschnitz C., Schuhmann M.K., Stegner D., Wultsch T., Eilers J., Meuth S.G., Stoll G. (2009). STIM2 Regulates Capacitive Ca^2+^ Entry in Neurons and Plays a Key Role in Hypoxic Neuronal Cell Death. Sci. Signal..

[212] Oh M.R., Lee K.J., Huang M., Kim J.O., Kim D.H., Cho C.-H., Lee E.H. (2017). STIM2 Regulates Both Intracellular Ca^2+^ Distribution and Ca^2+^ Movement in Skeletal Myotubes. Sci. Rep..

[213] Rana A., Yen M., Sadaghiani A.M., Malmersjö S., Park C.Y., Dolmetsch R.E., Lewis R.S. (2015). Alternative Splicing Converts STIM2 from an Activator to an Inhibitor of Store-Operated Calcium Channels. J. Cell. Biol..

[214] Miederer A.-M., Alansary D., Schwär G., Lee P.-H., Jung M., Helms V., Niemeyer B.A. (2015). A STIM2 Splice Variant Negatively Regulates Store-Operated Calcium Entry. Nat. Commun..

[215] Kim K.M., Rana A., Park C.Y. (2019). Orai1 Inhibitor STIM2β Regulates Myogenesis by Controlling SOCE Dependent Transcriptional Factors. Sci. Rep..

[216] Böhm J., Laporte J. (2018). Gain-of-Function Mutations in STIM1 and ORAI1 Causing Tubular Aggregate Myopathy and Stormorken Syndrome. Cell Calcium.

[217] Eberstein A., Goodgold J. (1968). Slow and Fast Twitch Fibers in Human Skeletal Muscle. Am. J. Physiol.—Leg. Content.

[218] Schiaffino S., Rossi A.C., Smerdu V., Leinwand L.A., Reggiani C. (2015). Developmental Myosins: Expression Patterns and Functional Significance. Skelet. Muscle.

[219] Calderón J.C., Bolaños P., Caputo C. (2010). Myosin Heavy Chain Isoform Composition and Ca(^2+^) Transients in Fibres from Enzymatically Dissociated Murine Soleus and Extensor Digitorum Longus Muscles. J. Physiol..

[220] Luff A.R., Atwood H.L. (1971). Changes in the Sarcoplasmic Reticulum and Transverse Tubular System of Fast and Slow Skeletal Muscles of the Mouse during Postnatal Development. J. Cell. Biol..

[221] McCarl C.-A., Picard C., Khalil S., Kawasaki T., Röther J., Papolos A., Kutok J., Hivroz C., LeDeist F., Plogmann K. (2009). ORAI1 Deficiency and Lack of Store-Operated Ca^2+^ Entry Cause Immunodeficiency, Myopathy, and Ectodermal Dysplasia. J. Allergy Clin. Immunol..

[222] Boncompagni S., Pecorai C., Michelucci A., Pietrangelo L., Protasi F. (2021). Long-Term Exercise Reduces Formation of Tubular Aggregates and Promotes Maintenance of Ca^2+^ Entry Units in Aged Muscle. Front. Physiol..

[223] Agbulut O., Destombes J., Thiesson D., Butler-Browne G. (2000). Age-Related Appearance of Tubular Aggregates in the Skeletal Muscle of Almost All Male Inbred Mice. Histochem. Cell. Biol..

[224] Schiaffino S., Reggiani C. (1996). Molecular Diversity of Myofibrillar Proteins: Gene Regulation and Functional Significance. Physiol. Rev..

[225] Dos Santos M., Backer S., Saintpierre B., Izac B., Andrieu M., Letourneur F., Relaix F., Sotiropoulos A., Maire P. (2020). Single-Nucleus RNA-Seq and FISH Identify Coordinated Transcriptional Activity in Mammalian Myofibers. Nat. Commun..

[226] Petrany M.J., Swoboda C.O., Sun C., Chetal K., Chen X., Weirauch M.T., Salomonis N., Millay D.P. (2020). Single-Nucleus RNA-Seq Identifies Transcriptional Heterogeneity in Multinucleated Skeletal Myofibers. Nat. Commun..

